# Supplementary material for: 4D reconstruction of murine developmental trajectories using spherical harmonics
Source: Dev Cell. 2022 Sep 12;57(17):2140–2150.e5. doi: 10.1016/j.devcel.2022.08.005 (PMC9481268; doi:10.1016/j.devcel.2022.08.005)
Supplement: Document S1. Figures S1–S5 [file mmc1.pdf]

Developmental Cell, Volume *57*

Supplemental information

4D reconstruction of murine developmental trajectories using spherical harmonics

Giovanni Dalmasso, Marco Musy, Martina Niksic, Alexandre Robert-Moreno, Claudio Badía-Careaga, Juan Jose Sanz-Ezquerro, and James Sharpe

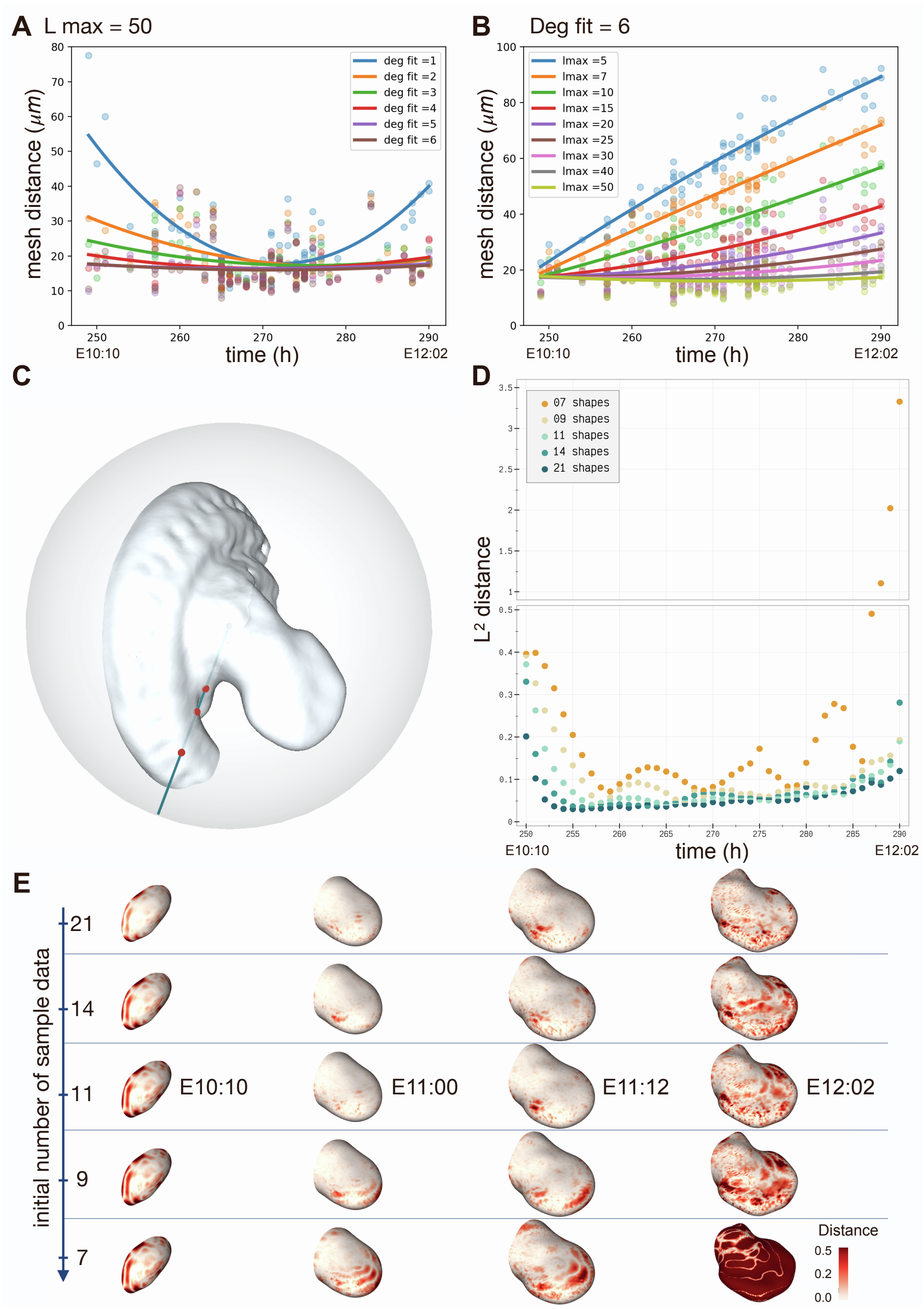

Figure S1: Assessing the degree of spherical harmonics expansion, related to Figure 1.

A - Comparison of different reconstructions according to mesh distances fixing the value of $l_{\max} = 50$ and varying the degree of the spline (from 1 to 6) interpolating the spherical harmonics coefficients.

B - Comparison of different reconstructions according to mesh distances fixing the degree's value of the spline interpolating the spherical harmonics coefficients and the value of $l_{\max}$ (5, 7, 10, 15, 20, 25, 30, 35, 40, 45, 50).

C - Representative example of a surface intersected three times (red dots) by a radius of a sphere which circumscribes it.

D - L^2 distance between sets of reconstructed and original surfaces reducing the total number of the initial set of data (i.e: 21, 14, 11, 9, 7).

E - Sets of reconstructed surfaces reducing the total number of the initial set of data (i.e: 21, 14, 11, 9, 7) at four different developmental stages (i.e: E10:10, E11:01, E11:12, E12:10). The color map shows the distance between the reconstructed meshes and the original data.

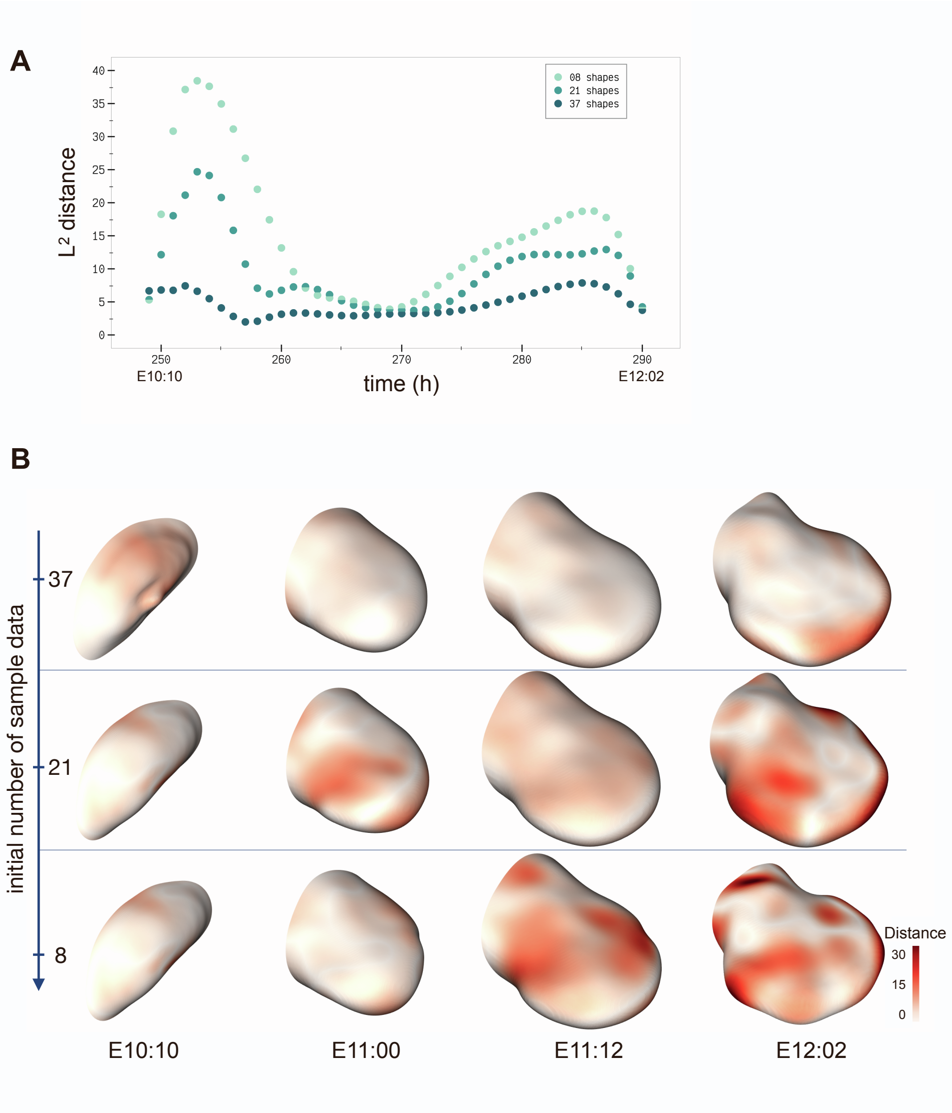

Figure S2: Assessing the degree of spherical harmonics expansion, related to Figure 1.
A - L^2 distance between sets of reconstructed and original surfaces reducing the total number of the initial set of data (i.e: 37, 21, 8).
B - Sets of reconstructed surfaces reducing the total number of the initial set of data (i.e: 37, 21, 8) at four different developmental stages (i.e: E10:10, E11:01, E11:12, E12:10). The color map shows the distance between the reconstructed meshes and the original data.

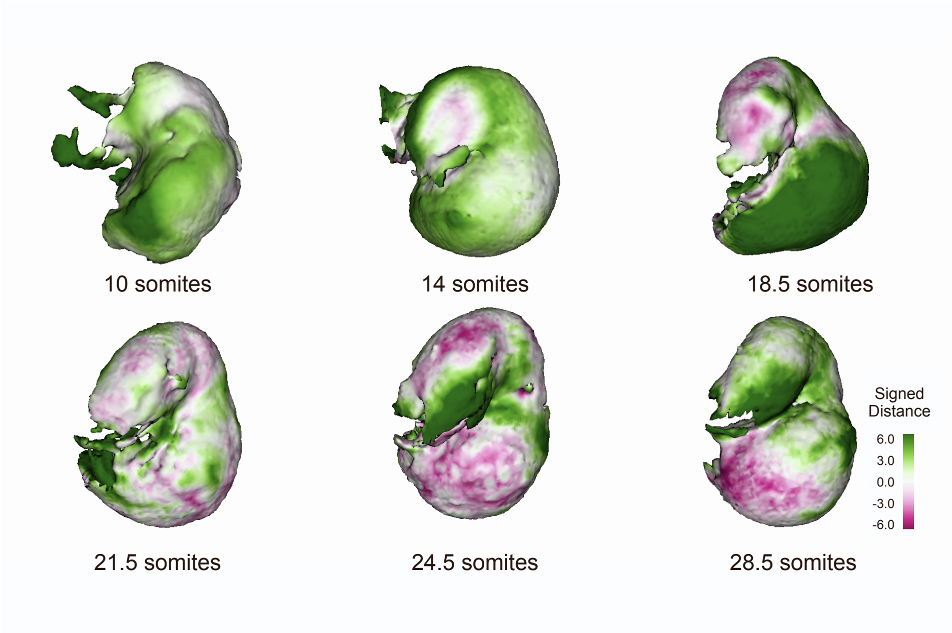

Figure S3: Precision of the reconstruction, related to Figure 5.
The heart reconstruction at representative developmental time points color map representing the signed distance between the reconstruction and the original surface.

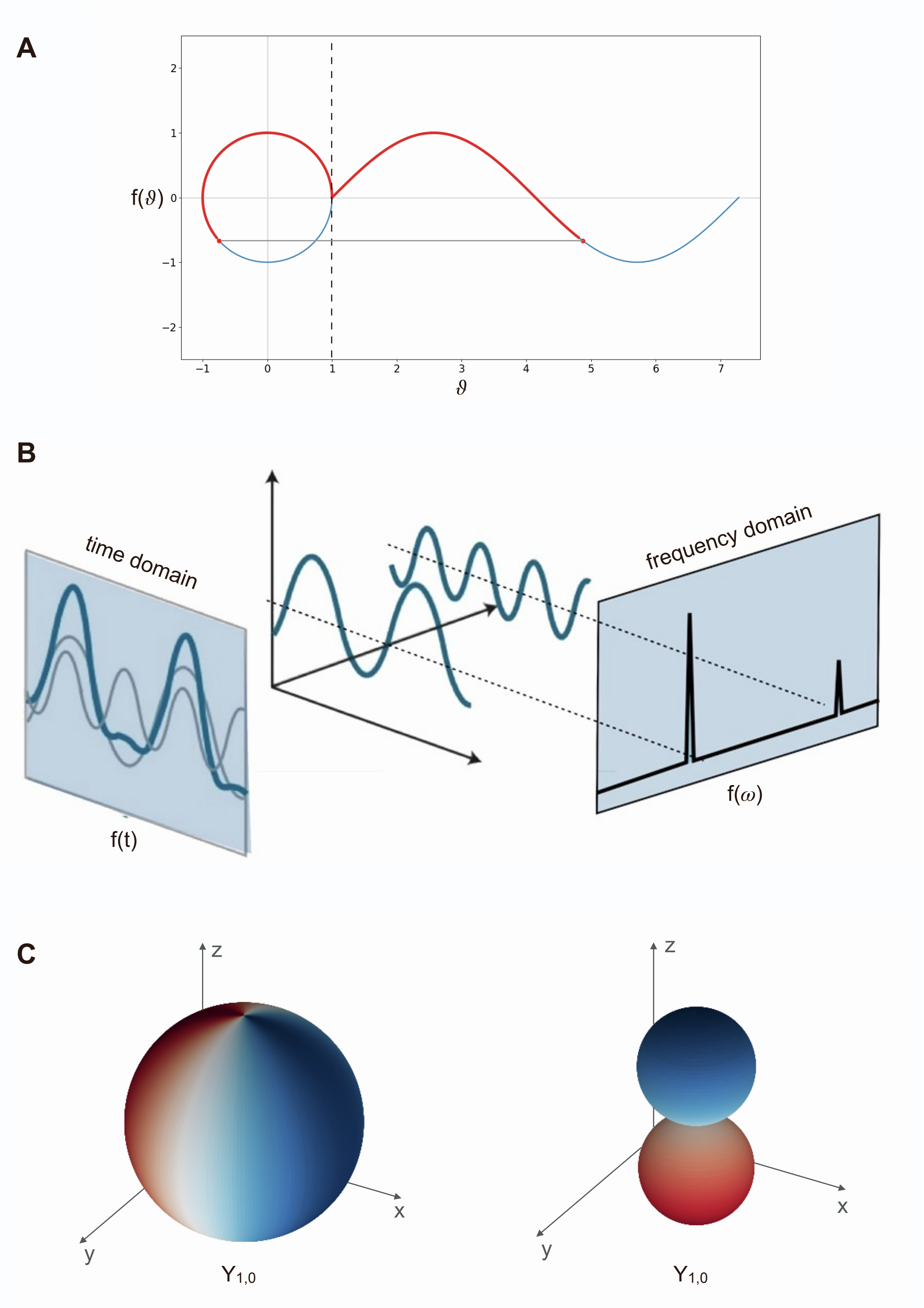

Figure S4: Spherical harmonics and Fourier expansion, related to STAR Methods.
A Representation of the function $\sin\vartheta$ and its projection onto the unit circle.
B Schematic representation of a function $f(t)$ in the time domain, its expression given by the sum of basis functions and its Fourier transform $f(\omega)$ in the frequency domain (*modified from* Gendler (2017)).
C Two equivalent representations of the spherical harmonic Y_1^0 of degree $l = 1$ and order $m = 0$.

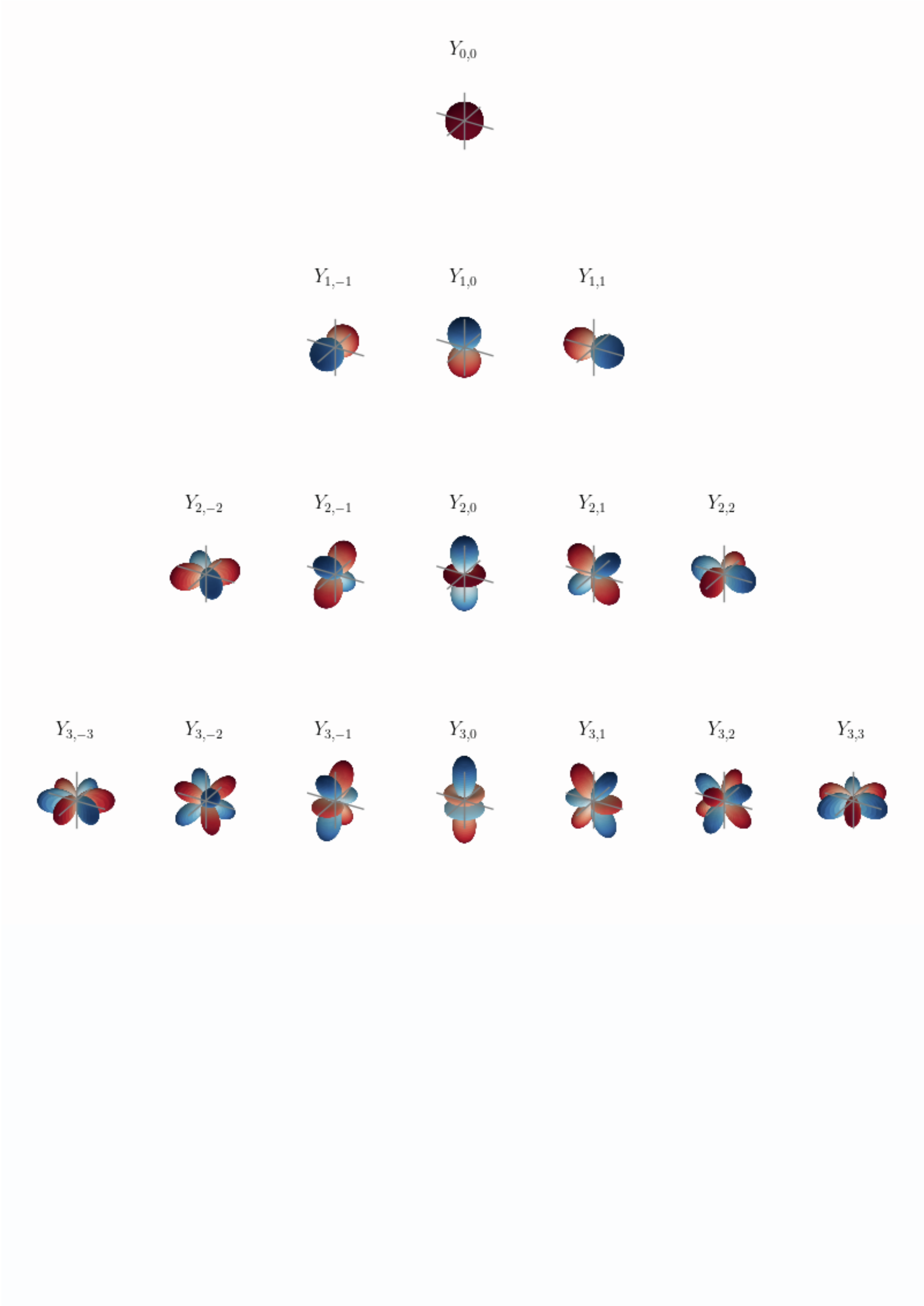

Figure S5: Hierarchical tree of spherical harmonics expansion, related to STAR Methods.
Representations of spherical harmonic functions for $l = 0, 1, 2, 3$.
