# Supplementary material for: 4D reconstruction of murine developmental trajectories using spherical harmonics
Source: Dev Cell. 2022 Sep 12;57(17):2140–2150.e5. doi: 10.1016/j.devcel.2022.08.005 (PMC9481268; doi:10.1016/j.devcel.2022.08.005)
Supplement: Document S2. Article plus supplemental information [file mmc2.pdf]

Technology

Developmental Cell

# 4D reconstruction of murine developmental trajectories using spherical harmonics

## Graphical abstract

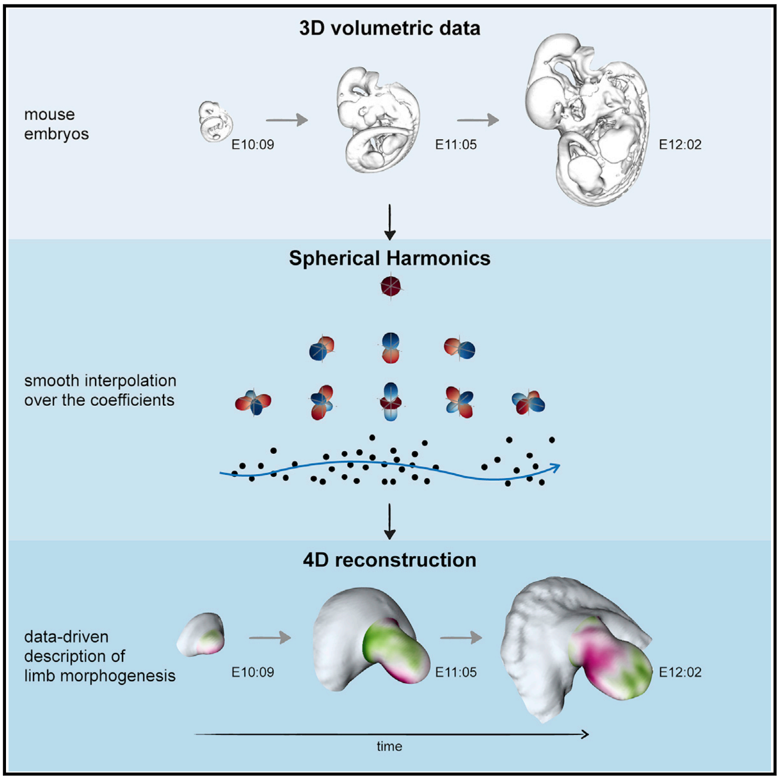



## Authors

Giovanni Dalmasso, Marco Musy, Martina Niksic, Alexandre Robert-Moreno, Claudio Badía-Careaga, Juan Jose Sanz-Ezquerro, James Sharpe

## Correspondence

giovanni.dalmasso@embl.es (G.D.), james.sharpe@embl.es (J.S.)



## In brief

Continuous time-lapse imaging of developing mammalian organs is not yet possible. Dalmasso et al. propose a computer-based approach to recreate a continuous evolution in time and space from a set of 3D volumetric images using spherical harmonics. The result allows for a data-driven quantitative 4D description of limb and heart morphogenesis.


## Highlights

- Computer-based method recreating a 3D plus time evolution of a set of volumetric images
- Technique based on the interpolation of the coefficients of spherical harmonics
- Data-driven quantitative 4D description of limb and heart morphogenesis
- Quantitatively reliable baseline description of organ development

Dalmasso et al., 2022, Developmental Cell *57*, 2140–2150
September 12, 2022 © 2022 The Authors. Published by Elsevier Inc.
https://doi.org/10.1016/j.devcel.2022.08.005

Technology

# 4D reconstruction of murine developmental trajectories using spherical harmonics

Giovanni Dalmasso,[1,6,*] Marco Musy,[1] Martina Niksic,[2] Alexandre Robert-Moreno,[1] Claudio Badía-Careaga,[3] Juan Jose Sanz-Ezquerro,[3,4] and James Sharpe[1,5,*]
[1]European Molecular Biology Laboratory (EMBL-Barcelona), 08003 Barcelona, Spain
[2]Centre for Genomic Regulation (CRG), 08003 Barcelona, Spain
[3]Centro Nacional de Investigaciones Cardiovasculares (CNIC), 28029 Madrid, Spain
[4]Centro Nacional de Biotecnologia (CSIC Madrid), 28049 Madrid, Spain
[5]Institució Catalana de Recerca i Estudis Avançats (ICREA), 08010 Barcelona, Spain
[6]Lead contact
*Correspondence: giovanni.dalmasso@embl.es (G.D.), james.sharpe@embl.es (J.S.)
https://doi.org/10.1016/j.devcel.2022.08.005

## SUMMARY

Normal organogenesis cannot be recapitulated *in vitro* for mammalian organs, unlike in species including *Drosophila* and zebrafish. Available 3D data in the form of *ex vivo* images only provide discrete snapshots of the development of an organ morphology. Here, we propose a computer-based approach to recreate its continuous evolution in time and space from a set of 3D volumetric images. Our method is based on the remapping of shape data into the space of the coefficients of a spherical harmonics expansion where a smooth interpolation over time is simpler. We tested our approach on mouse limb buds and embryonic hearts. A key advantage of this method is that the resulting 4D trajectory can take advantage of all the available data while also being able to interpolate well through time intervals for which there are little or no data. This allows for a quantitative, data-driven 4D description of mouse limb morphogenesis.

## INTRODUCTION

Progress in imaging technology has been central to understanding morphogenesis, which is an intrinsically 3D and dynamical process. In the case of externally developing organisms, such as *Drosophila* and zebrafish, it is now possible to image *in vivo* whole-embryo development at a cellular level, up to and including the later organ-forming stages (Tomer et al., 2012; Royer et al., 2016). However, for more complex animal models (e.g., mouse embryogenesis), it is still not possible to observe organogenesis in real time, due to the limitations of *in vitro* culture techniques. The dynamics of early post-implantation mouse embryogenesis have been successfully imaged in great detail (McDole et al., 2018); however, embryo culture beyond E10.5 is not robust enough to recapitulate the full development of complex organs—largely due to the lack of blood flow. *In vivo*/*in utero* imaging techniques (such as MRI) do not yet provide sufficient spatial resolution to capture the morphogenesis of organs, and our understanding of complex mammalian organogenesis is therefore largely derived from capturing series of *ex vivo* and static 3D images, often using mesoscopic imaging techniques such as optical projection tomography (Sharpe et al., 2002) (Figure 1A), light sheet imaging (Huisken et al., 2004), or *ex vivo* MRI (Wong et al., 2012, 2014).

An important remaining challenge is to integrate these series of static 3D datasets into a smooth, continuous and 4D trajectory that realistically models the morphogenesis of the organ in question. There have been diverse attempts to recapitulate the development of a full mouse embryo. Among others, Wong et al. (2015) used 3D optical projection tomography (OPT) images taken at six developmental time points (between E11.5 and E14.0), to create a 4D model of mouse embryo growth. Specifically, the outcome was the result of spline fitting and interpolating over time the displacement vectors of homologous points of the 3D voxel images. While this model provided a global overview of mouse embryo development, it was not able to accurately recreate smooth shape trajectories for all the organs. In particular, the registration of the voxel data was not able to align parts of the embryo whose positions are intrinsically variable over time, such as the limbs and the tail. Therefore, this method was not able to precisely reproduce the development of these particular mouse's structures. Indeed, the large degree of natural variation in samples represents a key challenge in the effort of recapitulating the growth of a mouse embryo or even a sub-part of it such as the limb.

Two embryos at the exact same age do not look identical, due to the intrinsic variation both in shape and in development. We therefore ideally need a technique which, on the one hand can take advantage of as much shape data as possible, while at the same time not giving excessive weight to any specific individual. In practice, this means a procedure in which each sample (i.e., each real limb) is able to influence the mean trajectory at ages both younger and older than itself, or, in other words, the final reconstruction needs to capture the right balance between


This is an open access article under the CC BY license (http://creativecommons.org/licenses/by/4.0/).

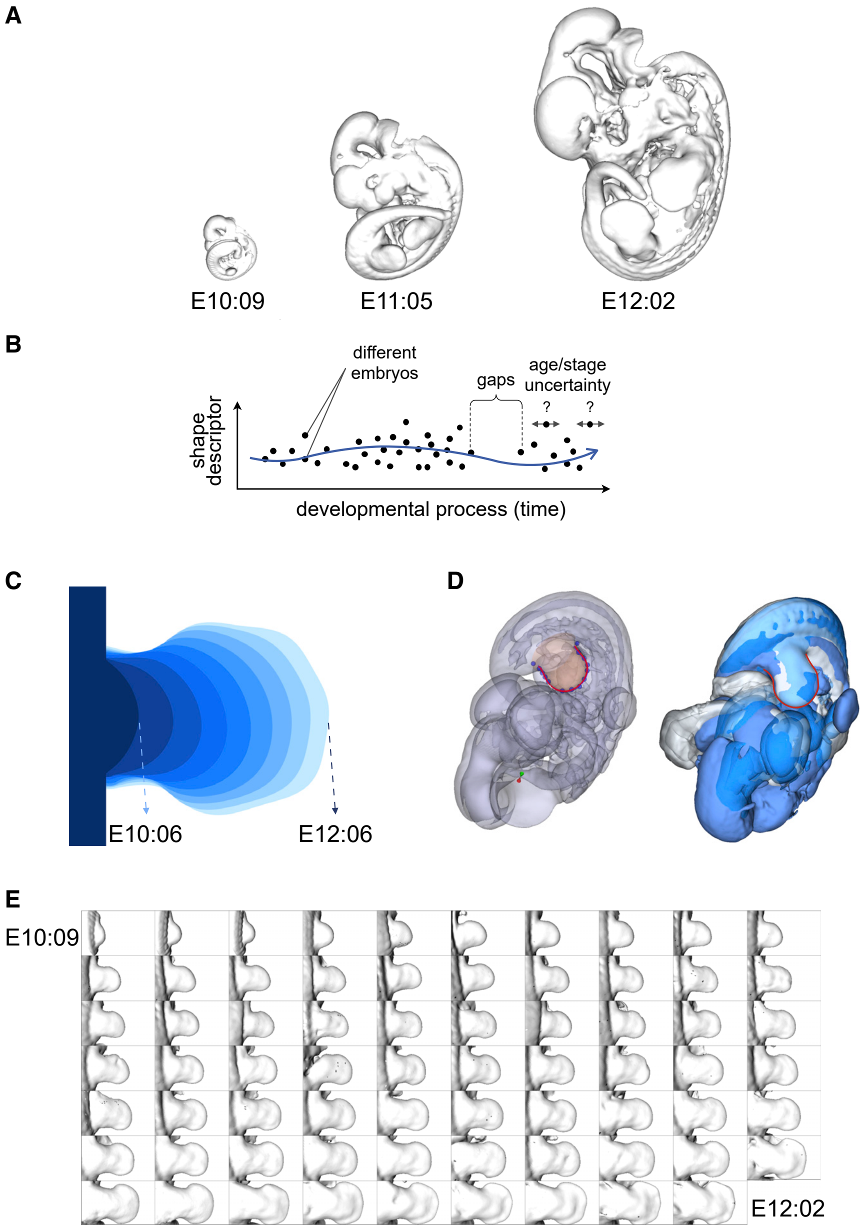


**Figure 1. Morphological growth of the mouse hind limb**

(A) Three mouse embryos at different developmental stages: E10:09, E11:05, and E12:02.

(B) Graphical illustration of a smooth trajectory taking into account all the different characteristics of the dataset.

(C) Two-dimensional interpolation of mouse limb from E10:06 to E12:06 at 6-h intervals.

(D) Alignment of three embryos at the same developmental stage according to their right hind limbs shown with transparency (left) and as surface in solid color (right). The red line represents the output of the staging system (Musy et al., 2018).

(E) Dataset of mouse right hind limbs used in this study ordered according to their developmental stage from E10:09 to E12:02.

the overall shape trends across the whole dataset, and the actual details of each limb. Another challenge is that the shape complexity of an organ increases during development. We need a method that performs some degree of dimensionality reduction, but without losing the more complex features of the older stages (Figure 1B).

Spherical harmonics have been known since 1782 (Laplace, *Mécanique Céleste*) and form a natural basis for describing how a scalar quantity varies on the surface of a sphere. They produce an orthogonal basis for mathematically describing a 3D shape and provide a compact parametric representation of it. Spherical harmonics have been widely used in different fields and, in recent years, also in biology. One of their main use has been to characterize, among others, the shapes of cells and organs (Styner et al., 2006; Morishita et al., 2017; Medyukhina et al., 2020), deformations and movements of tissues in an easier and more robust way (Khairy et al., 2018). Moreover, spherical harmonics expansion has been also applied to mesh refinements (Lai et al., 2009). The expansion of a function in spherical harmonics is characterized by a finite set of coefficients, which multiply the set of functions constituting the orthogonal basis. These coefficients encode the information of the original function; therefore, the higher the degree (i.e., the number of coefficients used) the more accurate the representation. Among other properties, one important feature that makes spherical harmonics particularly convenient is that the degree of the expansion represents the level of detail that is experimentally desirable. Therefore, limiting the number of coefficients of the series will not cause a loss of the main characteristic of the shape represented. Previous studies using spherical harmonics have considered collections of objects (both surfaces or volumes) often analyzed for the purpose of quantifying the differences or similarities between the sets. We are only aware of one previous use of spherical harmonics to characterize a changing morphology over time—in the chick embryo (Morishita et al., 2017). We therefore propose and demonstrate an approach to describe the evolution in time and space of mouse limbs both from volumetric and surface data.

A 2D trajectory of limb bud shape change has previously been created (not using spherical harmonics) and has been developed into a method to estimate the embryonic stage of a limb bud to a high temporal resolution (Musy et al., 2018; Figure 1C). This work, although only in 2D, allows us to have a reference to align in space and time mouse embryos according to a chosen limb (Figure 1D). Here, we develop and demonstrate two different versions of this approach for the case of limb development. We believe this approach has a widespread applicability in developmental biology, to provide a quantitatively reliable baseline

description of organ development—something surprisingly absent in the field so far—and so we also demonstrate its use on 3D images of another mouse organ, the developing heart.

## DESIGN

Our understanding of morphogenetic processes has increased in the last years due to the progress in image technologies. While it is now possible to image *in vivo* externally developing organisms, such as *Drosophila* and zebrafish, at a cellular level (Tomer et al., 2012; Royer et al., 2016), it is still unfeasible to observe organogenesis in real time for more complex animal models (e.g., mouse embryogenesis), due to the limitations of *in vitro* culture techniques. Additionally, the current available *in vivo/ in utero* imaging methods are not robust enough to recapitulate the full development of complex organs (McDole et al., 2018). Therefore, our notion of complex mammalian organogenesis is mainly derived from series of static 3D images (Huisken et al., 2004; Sharpe et al., 2002; Wong et al., 2012, 2014). The lack of possibilities to obtain continuous time-lapse imaging of developing mammalian organs, prompted us to develop a computer-based approach able to fill this gap and to recreate a continuous evolution in time and space of organ morphogenesis from a set of 3D volumetric images.

## RESULTS

Spherical harmonics, first investigated by Laplace in 1782 in his *Mécanique Céleste* are a set of expressions used to represent functions on the surface of the sphere $S^2$. They can be pictured as a higher-dimensional analogy of Fourier series, which form a complete basis for the set of periodic functions of a single variable (functions on a circle $S^1$).

The development of Fourier series in 19[th] century, made possible the solution of a wide variety of physical problems in rectangular domains, such as the solution of the heat equation or wave equation. This could be achieved by the expansion of functions in series of trigonometric expressions. Fourier series are indeed ideal for decomposing a periodic 2D signal in terms of sums of sines and cosines respectively weighted by a set of coefficients $a_n$ and $b_n$. Subsequently, to reconstruct the signal, it is essential to determine these coefficients in front of each term. Similarly, spherical harmonics use a set of functions depending this time on the radius, latitude, and longitude instead of abscissa and ordinate. In Fourier series, the order of each term would define the period (or wavelength) along the x axis while in spherical harmonics, two numbers are needed to take into account the two directions of waves: latitude and longitude. Whereas the trigonometric functions in a Fourier series represent the fundamental modes of vibration in a string, the spherical harmonics represent the fundamental modes of vibration of a sphere in approximately the same way. Many aspects of the theory of Fourier series could be generalized by taking expansions in spherical harmonics rather than trigonometric functions (see STAR Methods for more details).

Spherical harmonics represent therefore an ideal tool able to provide a compact parametric representation of complex 3D shapes (e.g., mouse limbs and hearts).

One key step in spherical harmonics expansion is to first map a surface onto a sphere. In the case of relatively simple surfaces, one possible solution is using as mapping the geometrical distance between the surface considered and a sphere which contains it. Specifically, all these distances represent a set of scalars onto the sphere which serve as a mapping that can subsequently be expanded in spherical harmonic. We applied this procedure to 69 surfaces of mouse limb buds imaged using OPT of mouse embryos previously ordered in time using the embryonic mouse ontogenetic staging system (eMOSS) (Musy et al., 2018). Expanding a function into a spherical harmonic series and reconstructing it from the spherical harmonic coefficients are the two essential procedures applied when operating with data on the sphere. However, in our work, instead of using the exact coefficients' values of the spherical harmonic series for reconstructing the original function, we interpolated them in time. In this way, the interpolation not only provides a spatial average estimate in the points where there is more than one surface, but it also produces a temporal estimate for the existent gaps due to missing data. The results show a reconstruction describing a continuous and smooth changing shape over space and time.

### Application on simple surfaces

An arbitrary surface, which can be mapped onto a sphere, can be expanded into a finite series of spherical harmonic terms and reconstructed back. The precision by which the reconstructed surface will resemble the original one depends on the number of coefficients of the expansion, the higher the number of coefficients the more precise the reconstruction.

As a dataset, we used a collection of mouse embryos polygonal surfaces extracted from OPT scans (Sharpe et al., 2002). All the embryos were staged, using the staging system already described, to be correctly positioned in time. Specifically, we obtained a set of 69 hind limbs from E10:09 to E12:22 (Figure 1E).

The eMOSS (Musy et al., 2018) provides us with a reference to align in space the limb buds. Afterward, the alignment of the sequence of limbs was refined using the iterative closest point (ICP) algorithm (Besl and McKay, 1992).

Since the limb is a rather simple surface, to map it onto a sphere, we used the geometrical distance between its surface and a sphere that circumscribes it (Figure 2A, top left). Along a given radius $r_j$, we measured the corresponding distance $d_j$ between a given surface $\mathbf{v} = (x, y, z)$ and the sphere. We used 500 radii along which computing these distances creating a set of 500 scalars $\mathbf{d}$ for each limb. On top on each sphere we obtain therefore a collection of sample points (equal to the number of radii) to which is associated a set of scalars $\mathbf{d}$ representing the distances between these points and the surface, as shown by the color heatmap in Figure 2A (top right). Moreover, the number of these sample points provides the resolution of the spherical mapping of the considered surfaces. Given the total number of limb surfaces $N$ we have therefore a set of scalars $\mathbf{d}_0, \ldots, \mathbf{d}_N$ which encodes the spherical mapping of each surface. Every spherical mapping can then be expanded in spherical harmonics.

A surface $\mathbf{v}$, mapped as $x(\vartheta,\varphi),\ y(\vartheta,\varphi),\ z(\vartheta,\varphi)$, can be represented in the form

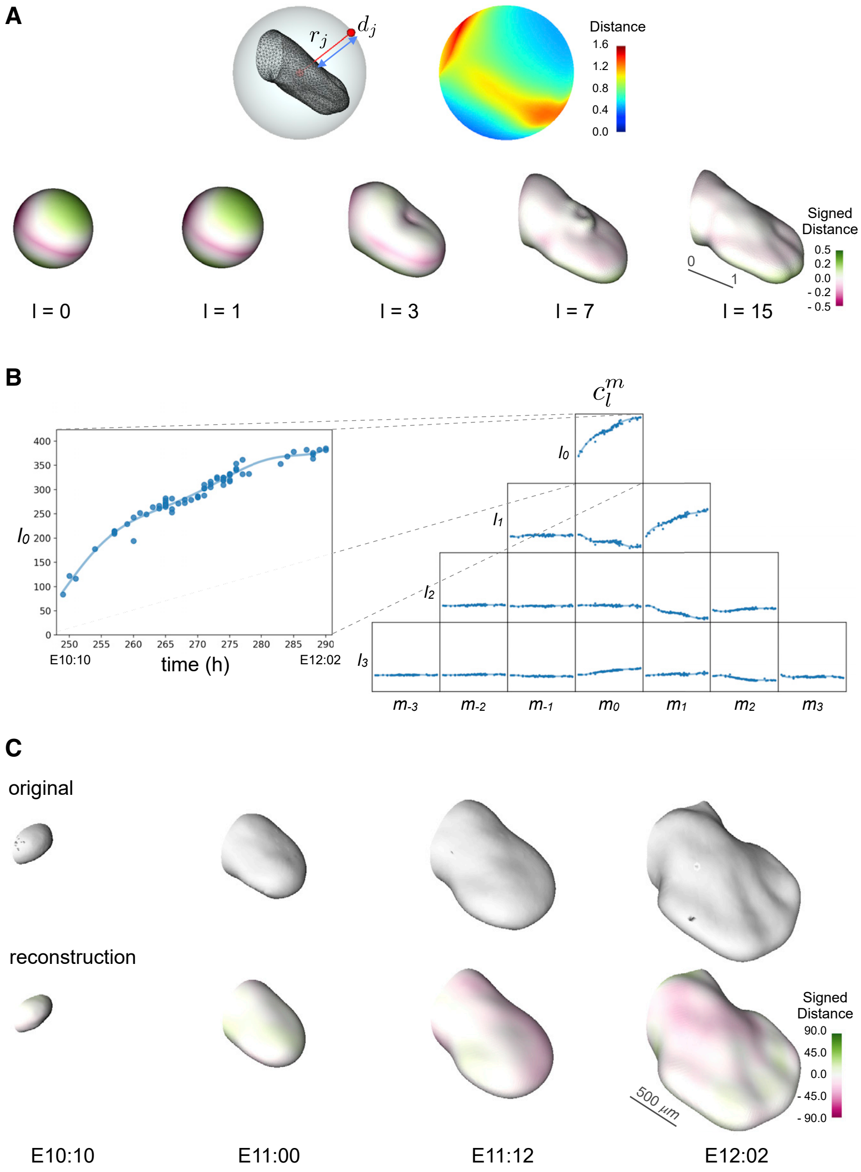


**Figure 2. Expansion using spherical harmonics**

(A) Mapping of a limb onto a sphere using geometrical distances (d) along the radii (r) between its surface and the sphere that circumscribes it (top left). Spherical mapping of a limb, the heatmap shows the distance between the points on top of the sphere and the surface of the limb (top right). Representation of the limb (in an a-dimensional reference system, scale bar corresponds to the unit length) using spherical harmonic expansion of different degrees, $l_{max}$ = 0, 1, 3, 7, 15 (bottom), and the color map represents the distance between the reconstruction and the original surface (see also Figure S1C).

(B) Splining in time over the spherical harmonics coefficients $c_l^m$. Here, the degrees $l$ = 0, 1, 2, 3, 4 are shown (see also Figures S1A and S1B).

(C) Comparison of four original data, out of the total of 69 mouse limbs used, at different developmental stages: E10:10, E11:00, E11:12, and E12:02 (top) with the corresponding limb growth reconstruction (bottom). The color map represents the signed distance between the reconstruction and the original surface and the scale bar corresponds to 500 µm (see also Figures S1D, S1E, and S2).

$$\mathbf{v}(\vartheta, \varphi) = \sum_{l=0}^{\infty} \sum_{m=-l}^{l} \mathbf{c}_l^m Y_l^m(\vartheta, \varphi) \quad \text{(Equation 1)}$$

where

$$Y_l^m(\vartheta, \varphi) = \sqrt{\frac{(2l+1)}{4\pi} \frac{(l-m)!}{(l+m)!}} P_l^m(\cos\vartheta)\ e^{im\varphi}, \quad \text{(Equation 2)}$$

$P_l^m$ are the associated Legendre polynomials and $\mathbf{c}_l^m = (c_{lx}^m, c_{ly}^m, c_{lz}^m)$, with $l = [0, \infty)$ and $m = [-l, +l]$, are the coefficients of the expansion. In particular, $\mathbf{c}_l^m$ assume the form:

$$\begin{array}{rccccc} \mathbf{c}_l^m = & & & c_0^0 & & \\ & & c_1^{-1} & c_1^0 & c_1^1 & \\ & c_2^{-2} & c_2^{-1} & c_2^0 & c_2^1 & c_2^2 \\ & & & \ldots & & \end{array} \quad \text{(Equation 3)}$$

In the practical use, the coefficients $\mathbf{c}_l^m$ of the expansion are truncated to a specific degree $l = l_{max}$, which defines the level of detail. Spherical harmonic expansion provides a unique description of a surface based on scalar coefficients. Using only the coefficients of the first degrees (i.e., $l_{max} = 0, 1$) provides as a result practically a sphere, with $l_{max} = 3$ the details of the reconstruction increase and adding more degrees ($l_{max} \geq 7$), it is possible to obtain a limb-like surface (Figure 2A, bottom). Moreover, the higher the order of the coefficient the less information it provides to the representation (i.e., in this reconstruction we are not interested in the finer morphological details). Therefore, every surface in our dataset could be represented by a finite set of coefficients of its corresponding spherical harmonic expansion. This means that we have the coefficients for each surface at its corresponding point in time. If we put together and represent all the coefficients of e.g., degree zero we will have a set of scalars evolving in time (Figure 2B, left), and the same happens for the subsequent degrees (Figure 2B, right). Instead of reconstructing each surface using the exact coefficients, we interpolated through them and used the interpolated values for the reconstruction (Figure 2C). In this way, the interpolated curve will give an average of the coefficients in the time points in which we have more than one surface and will fill the time gaps in the points in which there are no data. Since the aim of this work is to have the most precise reconstruction, fixing the degree $l_{max} = 50$, we compared the results (in terms of mesh distances) of using different degrees of polynomial interpolation (Figure S1A). In the same way, fixing the degree of interpolation

equal to 6, we compared the results of using different values of $l_{max}$ (Figure S1B). As shown in Figures S1A and S1B, the quality of the results reaches a plateau for an interpolation order of 6 and $l_{max} = 50$, which we adopted for the rest of this work. Using this approach for the coefficients of all degrees considered, it is possible to obtain a reconstruction in space and time of the dataset. In this reconstruction, each limb contributes the same to the average growing shape.

As a further improvement, we used this reconstruction to refine the alignment of the original dataset obtaining a better and more precise sequence of limbs (Figure 2C, top). Reapplying the method explained above in a way analogous to a bootstrapping process. With the new alignment of the data, we obtain an improved reconstruction of a growing limb bud trajectory (Figure 2C, bottom). This allows for a quantitative, data-driven 4D description of limb bud development across time and space (the result can only be well appreciated from watching Video 1, https://vedo.embl.es/fearless/#/limb).

The quality of the growing limb bud reconstruction obtained relies on the number of experimental shapes available and on its frequency in time. To achieve the optimal result, we used all the data available; however, to assess the robustness of the algorithm to temporal resolution, we performed an analysis to determine the minimum number of input shapes that provides a biological meaningful result. Since our set of input data is not uniformly distributed in time, for a first test, we use the same time course reconstruction that we obtained as ground truth. In this way we have a set of limbs with a uniform resolution of 1 h (from E10:10 to E12:02), and, most importantly, we have a control with which comparing the results of this test. Maintaining the shapes at the first and last time points, we subsequently reduced the number of input shapes by removing for each successive test more of the intermediate time points. In this manner, we applied our algorithm on a set of 21, 14, 11, 9, and 7 initial shapes and compared the results with the original reconstruction using the $L^2$ norm (we could not use a number lower than 7 since the order of the interpolation of the coefficients was 6). It is possible to notice that for a number of experimental shapes of 21, 14, and 11, the algorithm still produced reliable results (in terms of distance), whereas using 9 or 7 initial shapes decreased its efficiency dramatically (Figure S1D). From the $L^2$ distance between the results and the original data (Figure S1D), it is possible to see from quality of the reconstruction that it is not reliable for the entire time course. The result is more precise for the time points for which the original data are present, while it is not precise for the others, as shown by the behavior of the $L^2$ distance (Figure S1D). We can therefore conclude that using only 7 initial shapes, it is not enough for biologically reliable results. Additionally, regardless of the number of input shapes used, the most affected time points of the results were the initial and the final ones (Figure S1D). In Figure S1E, where the examples of reconstructed limbs (i.e., E10:10, E11:00, E11:12, and E12:02) are colored according to their distance with the original ones, it is possible to graphically see the results of the robustness of the study. While in all cases, the shapes obtained look like the original ones; analyzing the distances between them and the original data, it is possible to notice that the quality decreases by reducing the number of experimental shapes and, when using only an initial number of 7 shapes, the result is not biologically accurate, especially for the later time points where the shape of the limb is more complex.

After testing the algorithm on the reconstructed sequence of data, we also performed the same analysis on the original limb data. In the same way, as explained before, we fixed the shapes at the first and last time points, and we subsequently reduced the number of input shapes by removing at each step of every other shape. Since the original data cover a minor number of time points, and they are not uniformly distributed, we were able to apply our algorithm on a set of 37, 21, and 8 initial shapes and to compare the results with the original reconstruction using the $L^2$ norm. The result of this test is similar to the one before, giving less precise reconstructions by reducing the initial amount of data and showing that the most affected shapes are at the beginning and at the end of the time course (Figure S2A). An equivalent result can be seen in Figure S2B where examples of reconstructed limbs (i.e., E10:10, E11:00, E11:12, and E12:02) are colored according to their distance with the original ones. Moreover, it can be noticed that in this particular case, some shapes have some defects and do not look like real limbs, which can be appreciated, e.g., in the first limb (E10:10) of the first raw and in the two limbs (E11:00, E11:12) of the last raw of Figure S2B. These imperfections can derive from the fact that, reducing the initial number of data, some shapes start to have more weight on the reconstruction, and their defects, instead of being softened by the interpolation, are diffused along the reconstruction. Reducing the number of input shapes can therefore affect the interpolation in providing a smooth trajectory in which each limb contributes the same to the average growing shape.

### Adding the limb flank

In the previous section, we were able to recreate the growth in space and time of a mouse limb. In reality, however, the boundary between the limb and the rest of the embryo is rather arbitrary, and we therefore wanted to extend the analysis to include the flank of the embryo. Specifically, we extended the limb bud shape up to the mid-line of the mouse, as defined by the spinal column (Figure 3A). The new shape is now much more complex than before, displaying concavities (between the limb and the flank) that make it not projectable anymore onto a sphere. In particular, the key limitation of pure spherical harmonics arises when the radii used intersect the surface more than once (Figure S1C). Mapping methods for more complex surfaces and volumes have been extensively investigated (Brechbühler et al., 1995; Shen and Makedon, 2006; Lai et al., 2009; Shen et al., 2009; Yu et al., 2010), but nevertheless, these proposed methods are not suitable when multiple objects need to be consistently mapped onto the same reference since e.g., the portion of the sphere of the first one may not represent the same part of the second object.

To map our complex embryonic surface onto a sphere, we chose to make the use of volumetric data. For any arbitrary surface, it is possible to embed it into a volume and to compute the signed distances (SDs) over this volume from the input mesh (Baerentzen and Aanaes, 2005). The output is a volumetric dataset whose voxels contain the SD from the mesh. Inscribing this volume into a set of concentric spheres allow us to generate a set of scalar fields (i.e., SDs) on the sphere surface (Kazhdan et al., 2003; Skibbe et al., 2009). This allows us to expand the

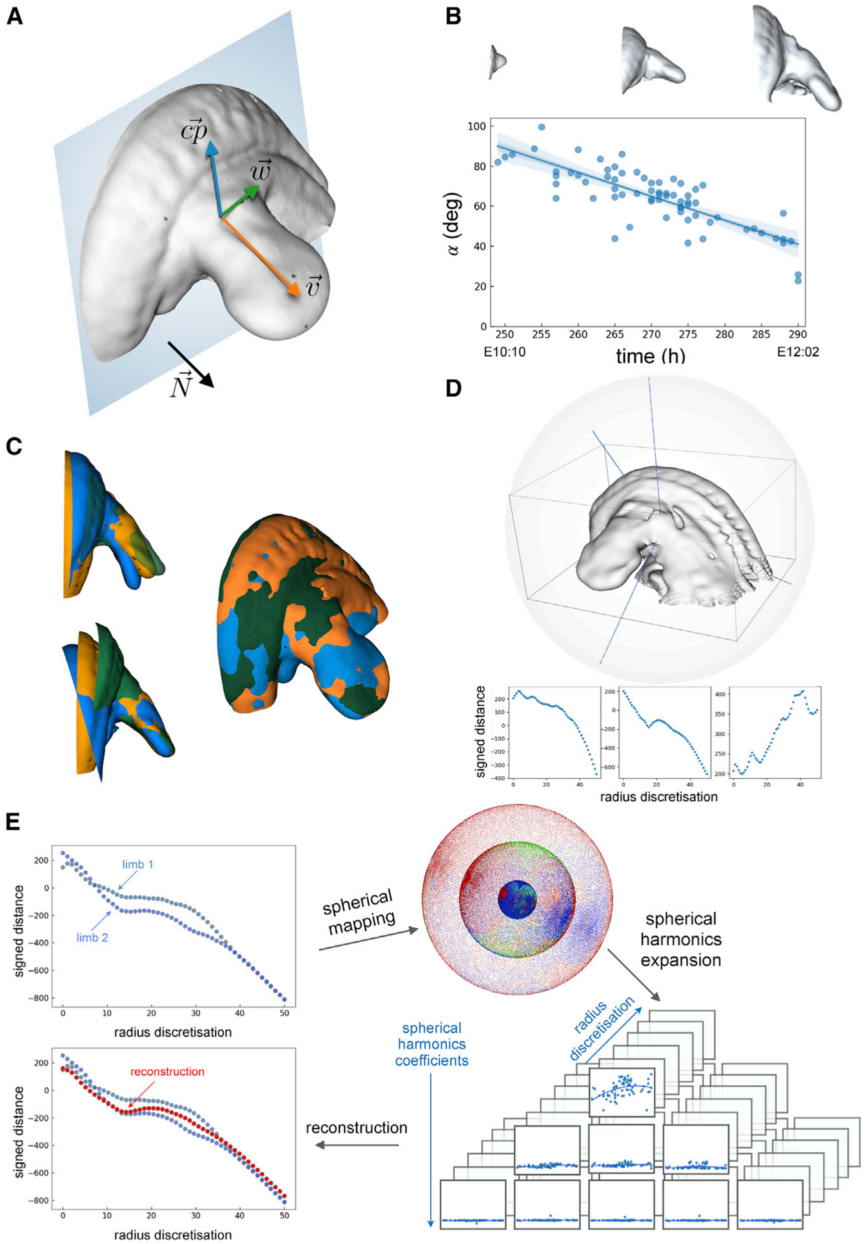


**Figure 3. Alignment and reconstruction using spherical harmonics**

(A) Triad $(\overrightarrow{cp}, \overrightarrow{w}, \overrightarrow{v})$ describing the orientations of the limb with respect to the normal $\overrightarrow{N}$ to a perpendicular plane.

(B) Variation in time of $\alpha = \angle(\overrightarrow{cp}, \overrightarrow{N})$ and its corresponding linear fitting with three representative limbs—E10:10, E11:25, and E12:02 (top).

(C) Alignment of three limbs with flank of the same developmental stage according to the flanks (top left) and to the limbs (bottom left), alignment according to the limbs and warping of the flanks (right).

(D) Example of signed distances computed on three different radii of a limb.

(E) Example of signed distance computed on the same radius of two limbs at the same developmental stage, their spherical mapping, coefficients of degree $l = 0,\ 1,\ 2$ of the spherical harmonics expansion, and comparison of the signed distance reconstruction (red dots) with the original ones (blue dots).

scalars of each sphere into spherical harmonics. In this way, we are able to have a spherical harmonic representation for any kind of surfaces. It is now possible to apply this procedure on the same dataset described before, but we can now consider not only the limb but also the flank (i.e., part of the mouse back) since we do not have any limitation on the kind of surface to be analyzed. Consequently, we can adopt the same concept used before of interpolating the spherical harmonics coefficients in time (over the developmental stages).

For a variety of reasons (external forces, natural variation, etc.) the limb buds of two embryos of the exact same age may not protrude from the flank at the same angle. However, a reasonable average shape cannot be calculated from two images that do not align. To create a reliable average trajectory of normal development, we need to make a judgment of which angle is the most normal, and we must then either select images that fit this average or alter the remaining datasets so that all limb buds and all flank regions can be well aligned. The first step was to measure the correct average angle. By defining an orthogonal triad of vectors $\mathbf{V} = (\overrightarrow{cp}, \overrightarrow{w}, \overrightarrow{v})$, it is possible to describe the movements and rotations of the limb in space. Moreover, considering a plane perpendicular to the limb, and therefore parallel to the flank, we can compute the angles between the triad $\mathbf{V}$ and a normal $\overrightarrow{N}$ to this plane (Figure 3A). Specifically, $\alpha = \angle(\overrightarrow{cp}, \overrightarrow{N})$ describes the vertical bending of the limb with respect to the flank, and thus, we can plot the angles for many embryos over time, which reveals a fairly linear decrease in this angle from about 90° at mE10:10 to about 40° at mE12:02 (Figure 3B). The second step was therefore to use a linear fit of $\alpha$ as a reference, and to apply a bending transform onto the shape data using "thin plate splines" (Bookstein, 1989), which is a non-linear transformation based on a physical analogy involving the bending of a rigid material. The limbs were not affected by this transformation, only the non-aligned flanks were bent. This operation ensures that all datasets reflect the average bending trajectory over time (Figure 3C).

To implement the idea mentioned above, we embedded each surface into a volume and computed the SDs over this volume from the input mesh (Baerentzen and Aanaes, 2005). The output is a volumetric dataset whose voxels contain the SD from the mesh. It is therefore possible to generate a scalar field by the SD from a mesh. Inscribing this volume into a sphere, it is possible to know the intensity of the SD along each chosen radius ($r_0, r_1, \ldots, r_n$) of this sphere making this mapping method

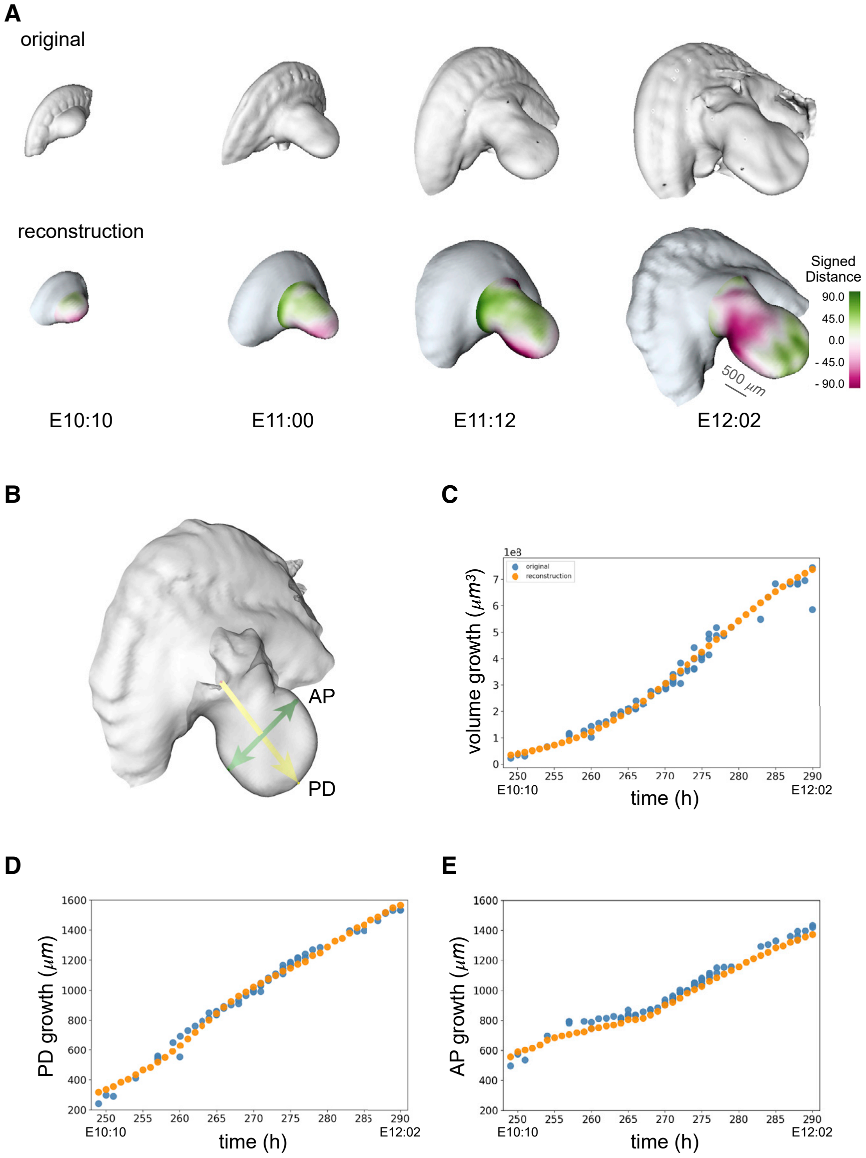


**Figure 4. Analysis and quantification**

(A) Comparison of four original data, out of the total of 69 mouse limbs used, at different developmental stages: E10:10, E11:00, E11:12, and E12:02 (top) with the corresponding limb growth reconstructions (bottom). The color map represents the signed distance between the reconstruction and the original surface and the scale bar corresponds to 500 μm.

(B) Representation of the proximodistal (PD) and anteroposterior (AP) axes of a limb.

(C) Volume growth comparison between original data (blue dots) and reconstructed shapes (orange dots).

(D) PD growth comparison between original data (blue dots) and reconstructed shapes (orange dots).

(E) AP growth comparison between original data (blue dots) and reconstructed shapes (orange dots).

theoretically applicable to any arbitrary surface (Figure 3D). Since each radius is discretized in $m = 50$ points, we have a set of $m$ concentric spheres on which the SDs are grouped according to their position along each radius. This represent a suitable condition that allows us to expand the scalars of each sphere into spherical harmonics.

With this mapping method, we obtained the $\mathbf{c}_l^m$ coefficients for each concentric spheres of every limb. Similar to that in the previous section, instead of reconstructing each volume using the exact coefficients $\mathbf{c}_l^m$, we first interpolated them over time. Subsequently, we used for the reconstruction the values of the interpolation, obtaining in this way one new limb for every time point (Figure 3E).

Every new limb is a volume object in which each voxel contains a scalar representing the reconstructed SD. Starting from this volume object, it is possible to additionally extract a corresponding surface. Using this method, we were able to create a time course reconstruction of a more complex object such as a mouse limb with its corresponding flank (Figure 4A).

The reconstruction in space and time of the growing limb consists of a smooth trajectory in developmental stages starting from E10:09 up to E12:02 (Figure 4A; Video 2, https://vedo.embl.es/fearless/#/limbflank). In order to know how accurately the result reproduces the 4D growth of a mouse limb, we directly compared it with the real data. To do so, we compared three distinctive characteristics of a mouse limb. Specifically, the total growth in the volume of the limb, its elongation along the proximodistal (PD) axis, and its widening along the anteroposterior (AP) axis (Figure 4B). For each reconstructed shape of a limb with its flank, the total volume was computed and compared with the volume of the original data at the same developmental stage (Figure 4C). In order to compare the limb elongation along the PD axis, we computed the geometrical distance between a fixed point (i.e., $[0,0,0]$) and the most distal point of the limb (Figure 4B), and we compared this value in the reconstructed and original data (Figure 4D). For confronting the enlargement of the limb along the AP axis the geometrical distance between the two furthest points in the AP plane of the limb "paddle" was determined and compared (Figure 4E).

The result represents the 4D growth of an "ideal" limb, which averages the common characteristics and features of all the limbs in the dataset. In particular, we are recreating the growing process starting from when the limb bud is just a small bump of tissue (E10:09) and finishing when it already shows a distinctive "paddle" shape of the autopod (E12:02). Additionally, the reconstruction is able to capture the correct biological scaling of a growing limb by accurately reproducing the volume growth shown in the data and its PD and AP axes evolution.

CellPress
OPEN ACCESS

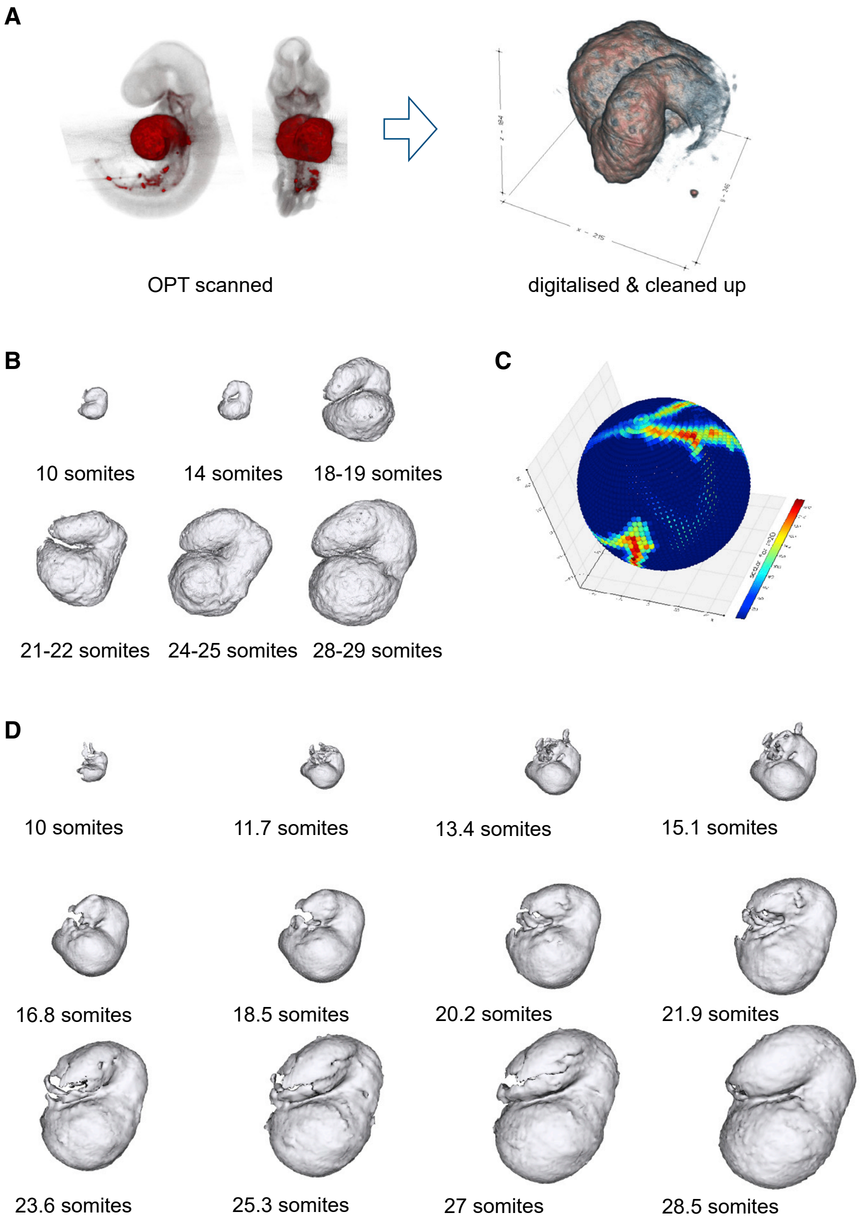


**Figure 5. Volumetric expansion and reconstruction using spherical harmonics**

(A) Representation of the data acquisition procedure: OPT scans of molecular marked mouse embryos, digitalization and cleaning of the data.

(B) Examples of heart volumetric data at different developmental stages (i.e., 10, 14, 18–19, 21–22, 24–25, and 28–29 somites), from the 27 used.

(C) Examples of scalar values of an 18–19 heart on one specific radius shell. Colors represent voxel intensities.

(D) The heart reconstruction at different developmental time points (see also Figure S2) .

## Application to the heart development

In order to determine the robustness of the method, we applied it on a completely different set of data, specifically volumetric mouse heart data. We used a total of 26 OPT scans of mouse embryos where the heart is distinguished from the rest of the embryonic tissue by means of a molecular marker (myosin heavy chain [MHC], as detected by antibody staining). The embryos were scanned by OPT and then processed to create surfaces representing only the MHC-expressing part of the heart (Figure 5A). We obtained mouse heart data at six different developmental stages, i.e., 10, 14, 18–19, 21–22, 24–25, and 28–29 somites (Figure 5B).

In this specific case, we considered the heart as a whole organ without the necessity of bending its surface. Therefore, differently from the previous section, it was possible to apply the method previously described directly on the volumetric data without the requirement of first converting them to surfaces and then using the SD.

As a first step, all the data were manually aligned using a 4 × 4 linear rigid transformation. Specifically, the registration was done using *Vedo* (Musy et al., 2022), which allows to interactively move and rotate the selected shapes, being in control of the displacements and distances in quantitative terms, and automatically save the aligned one and the matrix containing its registration. Afterward, similarly to the previous section, each heart was embedded into a sphere centered at the geometric center of the object, and the scalars, representing the voxel intensities, were computed along the radii of the sphere. The result is a set of intensities mapped on every concentric sphere from the radius discretization (Figure 5C). The scalar values mapped on these spheres were expanded into spherical harmonics. The coefficients $\mathbf{c}_l^m$ of the spherical harmonics were then interpolated over the developmental stages, and these interpolated values were used to reconstruct the heart trajectory.

The result, as in the case of the mouse limbs, is a smooth trajectory of a growing heart in space and time that takes into account the common characteristics and properties of all the samples in the dataset (Figures 5D and S3; Video 3—https://vedo.embl.es/fearless/#/heart). Thus, in spite of the fact that mouse limbs and mouse hearts are markedly different in shape, our algorithm demonstrates to be robust and produce a reliable reconstruction of the evolution in space and time of the original data in both cases. Moreover, this approach not only provides a quantitative basis for validating predictive models, but it also increases our understanding of morphogenetic processes from a purely geometrical point of view.

## DISCUSSION

Our understanding of limb development (and other examples of organogenesis) has been driven by a strong focus on molecular

and cellular activities. While modern biology has striven to make measurements of molecular data increasingly quantitative and complete (such as transcriptomics and single-cell approaches), surprisingly, an accurate quantitative description of the continuously changing morphology of the limb has not been created. Precision in such a description has perhaps not previously been required for a basic appreciation of mutant phenotypes, but as biology becomes more predictive and quantitative, accurate measurements of dynamic morphology will be essential. For example, the value of quantification for static 3D limb bud shape has already been shown to increase sensitivity in phenotyping studies (Martínez-Abadías et al., 2018), as well as enabling the detection of subtle but real alterations in gene expression patterns. Quantifying dynamic shape changes will also become important for mechanistic and predictive models of morphogenesis. For example, we previously used data-driven finite element modeling (FEM) to rule out a popular hypothesis about limb bud elongation (Boehm et al., 2010). By combing quantification of the changing 3D shape with accurate measurements of proliferation, we were able show that the proliferation gradient hypothesis cannot be correct because the model's predictions do not fit the true morphology.

While for some model species a 4D quantitative trajectory can be captured by direct time-lapse imaging, this is not true for mammalian organs. *In utero* imaging cannot provide high spatial resolution, and *in vitro* culture techniques do not support normal organogenesis. We are therefore left with the challenge of reconstructing a 4D dynamic process, from static snapshots. Here, we have developed and demonstrated a method to perform this task. It is based on the mathematical technique of spherical harmonics, which is a convenient method for dimensionality reduction of the data that can be spatially distributed on a sphere. We were able to use the coefficients of the spherical harmonics from 69 different limb buds, spanning an age range of mE10:09 to mE12:02 and spline through these values to create a smooth continuous 4D trajectory of normal mouse limb development. A simple, direct spherical harmonics method worked well on images just containing the limb bud itself, while a more complex volumetric version was required to interpolate a larger region of tissue that contained concavities (images which included a significant portion of the embryo trunk as well). A challenge for traditional landmark-based approaches, such as geometric morphometrics, is that the shape complexity dramatically increases from the beginning to the end of the trajectory, in other words, presenting a changing amount of useful shape information. The spherical harmonics approach we present here copes well with this challenge because of the intrinsic feature that coefficients of different degrees (different levels of detail) contribute independently to the overall shape description.

In conclusion, we have applied this method to both limb development and a sub-set of heart development (the MHC-expressing tissues) for the mouse embryo. It produces a smooth, continuous, and quantitative 4D description of their morphogenesis, including predictions for the gradual changes in lengths and volumes of the tissue. We believe it can be applied to many other developing organs and will be increasingly important as developmental biology becomes a more quantitative science and moves toward predictive mechanistic computer modeling of morphogenesis.

## Limitations of the study

In order to produce an accurate reconstruction, the original data need to be aligned in space before applying this method, either manually or automatically. Therefore, a poor initial alignment of the data will provide unreliable results. Additionally, in the case of the mouse limbs and hearts, it is possible to obtain a continuous and quantitative result with a reasonable small amount of spherical harmonics coefficients, more complex organs could require an higher amount of coefficients, affecting the speed of the code.

## STAR★METHODS

Detailed methods are provided in the online version of this paper and include the following:

- KEY RESOURCES TABLE
- RESOURCE AVAILABILITY
  - Lead contact
  - Materials availability
  - Data and code availability
- EXPERIMENTAL MODEL AND SUBJECT DETAILS
  - Animals
  - Husbandry conditions of experimental animals
  - Housing conditions of experimental animals
- METHOD DETAILS
  - Spherical Harmonics
  - Implementation
  - Whole-mount antibody staining
  - Optical projection tomography
- QUANTIFICATION AND STATISTICAL ANALYSIS
- ADDITIONAL RESOURCES

## SUPPLEMENTAL INFORMATION

Supplemental information can be found online at https://doi.org/10.1016/j.devcel.2022.08.005.

## ACKNOWLEDGMENTS

We thank Isaac Esteban and Miguel Torres for their invaluable discussions on shape comparison and spherical harmonics; Antoni Matyjaszkiewicz for his help with the webpage of the project; Isabel Romero Calvo for her precious suggestions on the graphical abstract and Carla Manzanas for the media coverage. This research was supported by core funding from EMBL and CRG, and also the ERC advanced grant SIMBIONT (project no. 670555) and a grant from the Fundacion La Marato TV3 (grant 082030). G.D. and M.M. were supported by the ERC advanced grant SIMBIONT (project no. 670555).

## AUTHOR CONTRIBUTIONS

G.D.: conceptualization, data curation, formal analysis, investigation, methodology, and writing – original draft; M.M.: investigation, methodology, and writing – review & editing; M.N., A.R.-M., C.B.-C. performed the experiments; J.J.S.E.: supervision, funding acquisition, and writing – review & editing; J.S.: conceptualization, supervision, funding acquisition, project administration, and writing – review & editing.

## DECLARATION OF INTERESTS

The authors declare no competing interests.

Received: December 16, 2021
Revised: May 2, 2022
Accepted: August 11, 2022
Published: September 1, 2022

## REFERENCES

Arfken, G.B., Weber, H.J., and Harris, F.E. (2013). Mathematical Methods for Physicists, Seventh Edition (Academic Press).

Baerentzen, J.A., and Aanaes, H. (2005). Signed distance computation using the angle weighted pseudonormal. IEEE Trans. Vis. Comput. Graph. *11*, 243–253. http://ieeexplore.ieee.org/document/1407857/.

Besl, P.J., and McKay, N.D. (1992). A method for registration of 3-D shapes. IEEE Trans. Pattern Anal. Mach. Intell. *14*, 239–256.

Boehm, B., Westerberg, H., Lesnicar-Pucko, G., Raja, S., Rautschka, M., Cotterell, J., Swoger, J., and Sharpe, J. (2010). The role of spatially controlled cell proliferation in limb bud morphogenesis. PLoS Biol. *8*, e1000420. https://journals.plos.org/plosbiology/article?id=10.1371/journal.pbio.1000420.

Bookstein, F.L. (1989). Principal warps: thin-plate splines and the decomposition of deformations. IEEE Trans. Pattern Anal. Mach. Intell. *11*, 567–585.

Brechbühler, C., Gerig, G., and Kübler, O. (1995). Parametrization of closed surfaces for 3-D shape description. Comput. Vision Image Underst. *61*, 154–170.

Dalmasso, G. (2022). FeARLesS: 4D reconstruction of developmental trajectories using spherical harmonics (Zenodo). https://doi.org/10.5281/zenodo.6962145.

Griffith, D.J. (2006). Introduction to Quantum Mechanics, Second Edition (Wiley).

Harris, C.R., Millman, K.J., van der Walt, S.J., Gommers, R., Virtanen, P., Cournapeau, D., Wieser, E., Taylor, J., Berg, S., Smith, N.J., et al. (2020). Array programming with NumPy. Nature *585*, 357–362. https://doi.org/10.1038/s41586-020-2649-2.

Hill, C. (2020). Learning Scientific Programming with Python (Cambridge University Press). https://www.cambridge.org/core/product/identifier/9781108778039/type/book.

Huisken, J., Swoger, J., Del Bene, F., Wittbrodt, J., and Stelzer, E.H. (2004). Optical sectioning deep inside live embryos by selective plane illumination microscopy. Science *305*, 1007–1009.

Kazhdan, M., Funkhouser, T., and Rusinkiewicz, S. (2003). Rotation invariant spherical harmonic representation of 3D shape descriptors. In Symposium on Geometry Processing: Proceedings of the 2003 Eurographics/ACM SIGGRAPH Symposium on Geometry Processing (The Eurographics Association), pp. 156–164.

Khairy, K., Lemon, W., Amat, F., and Keller, P.J. (2018). A preferred curvature-based continuum mechanics framework for modeling embryogenesis. Biophys. J. *114*, 267–277. https://doi.org/10.1016/j.bpj.2017.11.015. http://linkinghub.elsevier.com/retrieve/pii/S0006349517312444.

Kong, Q., Siauw, T., and Bayen, A. (2020). Python programming and numerical methods: a guide for engineers and scientists (Elsevier Science). https://books.google.es/books?id=cZ4LEAAAQBAJ.

Lai, S., Cheng, F., and Fan, F. (2009). Fast spherical mapping for genus-0 meshes. Lecture Notes in Computer Science (including subseries Lecture Notes in Artificial Intelligence and Lecture Notes in Bioinformatics). 5876 LNCS(PART 2), pp. 982–991. https://www.semanticscholar.org/paper/Fast-Spherical-Mapping-for-Genus-0-Meshes-Vegas-Nevada./4f2ae2bf99f1e78ae0e0ca0efb3bb60604cd93e1?p2df.

Lorensen, W.E., and Cline, H.E. (1987). Marching cubes: a high resolution 3D surface construction algorithm. SIGGRAPH Comput. Graph. *21*, 163–169.

Martínez-Abadías, N., Mateu Estivill, R., Sastre Tomas, J., Motch Perrine, S., Yoon, M., Robert-Moreno, A., Swoger, J., Russo, L., Kawasaki, K., Richtsmeier, J., and Sharpe, J. (2018). Quantification of gene expression patterns to reveal the origins of abnormal morphogenesis. eLife *7*, e36405. https://doi.org/10.7554/eLife.36405.001.

McDole, K., Guignard, L., Amat, F., Berger, A., Malandain, G., Royer, L.A., Turaga, S.C., Branson, K., and Keller, P.J. (2018). In toto imaging and reconstruction of post-implantation mouse development at the single-cell level. Cell *175*, 859.e33. 876.e33.

Medyukhina, A., Blickensdorf, M., Cseresnyés, Z., Ruef, N., Stein, J.V., and Figge, M.T. (2020). Dynamic spherical harmonics approach for shape classification of migrating cells. Sci. Rep. *10*, 6072. https://www.nature.com/articles/s41598-020-62997-7.

Morishita, Y., Hironaka, K.I., Lee, S.W., Jin, T., and Ohtsuka, D. (2017). Reconstructing 3D deformation dynamics for curved epithelial sheet morphogenesis from positional data of sparsely-labeled cells. Nat. Commun. *8*, 15. https://www.nature.com/articles/s41467-017-00023-7.pdf.

Musy, M., Flaherty, K., Raspopovic, J., Robert-Moreno, A., Richtsmeier, J.T., and Sharpe, J. (2018). A quantitative method for staging mouse embryos based on limb morphometry. Development *145*, dev154856. https://doi.org/10.1242/dev.154856.

Musy, M., Jacquenot, G., Dalmasso, G., Badger, C., de Bruin, B., and Sullivan, B. (2022). *vedo, a python module for scientific analysis and visualization of 3D objects and point clouds*. Zenodo. https://doi.org/10.5281/zenodo.2561401.

Poulenard, A., Rakotosaona, M.J., Ponty, Y., and Ovsjanikov, M. (2019). Effective rotation-invariant point CNN with spherical harmonics kernels. In Proceedings - International Conference on 3D Vision, 3DV 2019, *2019*, pp. 47–56. HAL Inria. https://hal.inria.fr/hal-02167454v2.

Royer, L.A., Lemon, W.C., Chhetri, R.K., Wan, Y., Coleman, M., Myers, E.W., and Keller, P.J. (2016). Adaptive light-sheet microscopy for long-term, high-resolution imaging in living organisms. Nat. Biotechnol. *34*, 1267–1278.

Sarkans, U., Gostev, M., Athar, A., Behrangi, E., Melnichuk, O., Ali, A., Minguet, J., Rada, J.C., Snow, C., Tikhonov, A., et al. (2018). The BioStudies database—one stop shop for all data supporting a life sciences study. Nucleic Acids Res. *46*, D1266–D1270. https://academic.oup.com/nar/article/46/D1/D1266/4561649.

Shafkat, I. (2021). Visual notes on spherical harmonics. https://irhum.github.io/blog/spharmonics.

Sharpe, J., Ahlgren, U., Perry, P., Hill, B., Ross, A., Hecksher-Sørensen, J., Baldock, R., and Davidson, D. (2002). Optical projection tomography as a tool for 3D microscopy and gene expression studies. Science *296*, 541–545. http://www.ncbi.nlm.nih.gov/pubmed/11964482.

Shen, L., Farid, H., and Mcpeek, M.A. (2009). Modeling three-dimensional morphological structures using spherical harmonics. Evolution *63*, 1003–1016. https://onlinelibrary.wiley.com/doi/pdf/10.1111/j.1558-5646.2008.00557.x.

Shen, L., and Makedon, F. (2006). Spherical mapping for processing of 3D closed surfaces. Image Vision Comput. *24*, 743–761. https://linkinghub.elsevier.com/retrieve/pii/S0262885606000485.

Skibbe, H., Wang, Q., Ronneberger, O., Burkhardt, H., and Reisert, M. (2009). Fast computation of 3D spherical Fourier harmonic descriptors—a complete orthonormal basis for a rotational invariant representation of three-dimensional objects. In 2009 IEEE 12th International Conference on Computer Vision Workshops, ICCV Workshops 2009 (IEEE Publications), pp. 1863–1869.

Styner, M., Oguz, I., Xu, S., Brechbühler, C., Pantazis, D., Levitt, J.J., Shenton, M.E., and Gerig, G. (2006). Framework for the statistical shape analysis of brain structures using SPHARM-PDM. Insight J. 242–250. http://www.ncbi.nlm.nih.gov/pmc/articles/PMC3062073/.

Tomer, R., Khairy, K., Amat, F., and Keller, P.J. (2012). Quantitative high-speed imaging of entire developing embryos with simultaneous multiview light-sheet microscopy. Nat. Methods *9*, 755–763.

Wieczorek, M.A., and Meschede, M. (2018). SHTools: tools for working with spherical harmonics. Geochem. Geophys. Geosyst. *19*, 2574–2592.

Wong, M.D., Dorr, A.E., Walls, J.R., Lerch, J.P., and Henkelman, R.M. (2012). A novel 3D mouse embryo atlas based on micro-CT. Development *139*, 3248–3256.

Wong, M.D., Maezawa, Y., Lerch, J.P., and Henkelman, R.M. (2014). Automated pipeline for anatomical phenotyping of mouse embryos using micro-CT. Development *141*, 2533–2541.

Wong, M.D., Van Eede, M.C., Spring, S., Jevtic, S., Boughner, J.C., Lerch, J.P., and Henkelman, R.M. (2015). 4D atlas of the mouse embryo for precise morphological staging. Development *142*, 3583–3591.

Yu, W., Ye, T., Li, M., and Li, X. (2010). Spherical harmonic decomposition for surfaces of arbitrary topology. ICCSE 2010 - 5th International Conference on Computer Science and Education, Final Program and Book of Abstracts (IEEE Publications), pp. 215–220.

CellPress
OPEN ACCESS

# STAR★METHODS

## KEY RESOURCES TABLE

| REAGENT or RESOURCE | SOURCE | IDENTIFIER |
|---|---|---|
| Antibodies | | |
| anti-Myosin heavy chain, sarcomere (MHC) antibody | Developmental Studies Hybridoma Bank | MF20; RRID: AB_1857203 |
| biotin goat anti-mouse IgG | Jackson ImmunoResearch | 115-066-071; RRID: AB_1655245 |
| streptavidin-Cy3 | Jackson ImmunoResearch | 016-160-084; RRID: AB_1948868 |
| Deposited data | | |
| Raw data | This paper; BioStudies database (Sarkans et al., 2018) | https://www.ebi.ac.uk/biostudies/studies/S-BIAD441 |
| Experimental models: Organisms/strains | | |
| Mouse embryos: C57Bl6/J | The Jackson Laboratory | C57Bl6/J |
| Software and algorithms | | |
| Python 3 | Python Software Foundation | https://www.python.org |
| eMOSS staging system | Musy et al., 2018 | https://limbstaging.embl.es |
| Vedo | | https://vedo.embl.es |
| FeARLesS | Dalmasso, 2022 | https://doi.org/10.5281/zenodo.6962145 |
| Skyscan | Bioptonics, MRC Technology | https://www.microphotonics.com |
| NRecon | Bioptonics, MRC Technology | https://www.microphotonics.com |
| Bioptonics Viewer | Bioptonics, MRC Technology | https://www.bioptonics.com |
| Other | | |
| Optical projection tomography (OPT) imaging | Sharpe et al., 2002 | N/A |

## RESOURCE AVAILABILITY

### Lead contact

Further information and requests for resources and reagents should be directed to and will be fulfilled by the lead contact, Giovanni Dalmasso (giovanni.dalmasso@embl.es)

### Materials availability

This study did not generate new unique reagents.

### Data and code availability

- All the original data (mouse limbs and hearts) used in this study, and all the mouse embryos data used in Figures 1A and 1D have been deposited in the BioStudies database (Sarkans et al., 2018) → https://www.ebi.ac.uk/biostudies/studies/S-BIAD441 and are publicly available as of the date of publication. Accession numbers are listed in the key resources table.
- All original code, publicly available as of the date of publication, has been deposited in a GitHub repository and can be found at → https://doi.org/10.5281/zenodo.6962145 (Dalmasso, 2022). DOIs are listed in the key resources table.
- Any additional information required to reanalyze the data reported in this paper is available from the lead contact upon request.

## EXPERIMENTAL MODEL AND SUBJECT DETAILS

### Animals

All animal work was performed according to the guidelines of the Committees on Ethics and Animal Welfare established at PRBB and CNIC, in accordance to Spanish and European laws. C57Bl6/J mouse embryos were collected at the indicated gestational stages. For a precise staging of limb buds, the eMOSS staging system was used (Musy et al., 2018), and for hearts, pairs of somites were counted. Embryos were dissected in cold PBS and fixed overnight in 4% PFA at 4° C.

### Husbandry conditions of experimental animals

The animal facility from the PRBB is fully accredited by the International Association for Assessment and Accreditation of Laboratory Animal Care (AAALAC).

### Housing conditions of experimental animals

At the Barcelona Biomedical Research Park (PRBB) animal facility, accredited by AAALAC (The Association for Assessment and Accreditation of Laboratory Animal Care International), animals were regularly monitored for any health concerns. Mice were kept in individually ventilated cages (IVCs; Tecniplast, Italy) at a temperature of 20° C to 24° C, humidity of 40% to 60% and a 12/12-hour light/dark cycle with the lights on at 7:30 AM. Cages contained bedding of large fibrous particles (Souralit 1035, Bobadeb S.L, Spain) and tissue papers and cardboard tubes as cage enrichment. Animals had free access to water and irradiated RM3 diet (Special Diet Services, England. All materials, including IVCs, lids, feeders, bottles, bedding, and water were autoclaved before use.

At Centro Nacional de Investigaciones Cardiovasculares (CNIC) mice were housed in a specific pathogen-free facility under a 12h light/12h dark cycle, at 20°-24° C temperature and relative humidity 60%, with water and food available ad libitum. Animals were monitored daily for well-being.

## METHOD DETAILS

### Spherical Harmonics

Spherical harmonics are a set of functions that form a basis that can be used to represent functions on the surface of the sphere $S^2$. They can be considered as a higher-dimensional analogy to Fourier series, which constitute a complete basis for the set of a single variable, i.e. functions on the circle $S^1$. While Fourier series are a suitable decomposition for functions in cartesian coordinates, spherical harmonics allow to represent any function, defined either in spherical coordinates, as a sum of a set of basis functions.

Since spherical harmonics are defined as the eigenfunctions of the angular part of the Laplacian ($\nabla \cdot \nabla,\ \nabla^2$ or $\Delta$) in three dimensions, they are particularly suitable in representing solutions to partial differential equation (PDE) in which the Laplacian appears. The Laplacian is a central part of significant physical equations, such as the heat equation, Schrödinger equation, wave equation, Poisson equation, and Laplace equation, which are ubiquitous in gravity, electromagnetism/radiation, and quantum mechanics. Therefore, spherical harmonics are fundamental for representing physical quantities of interest in these domains, most notably the orbitals of the hydrogen atom in quantum mechanics (Griffith, 2006). Additionally, spherical harmonics have recently been used in the field of machine learning (Poulenard et al., 2019).

#### *Brief history*

Spherical harmonics were first explored in relation with the Newtonian potential of Newton's law of universal gravitation in three dimension. Pierre-Simon de Laplace determined in his *Mécanique Céleste* (1782) that the gravitational potential $\mathrm{R}^3 \rightarrow \mathrm{R}$ at a point $\mathbf{x}$ associated to a set of point masses $m_j$ locate at points $\mathbf{x}_j$ was given by

$$V(\mathbf{x}) = \sum_j \frac{m_j}{|\mathbf{x}_j - \mathbf{x}|}. \qquad \text{(Equation 4)}$$

Each term in Equation 4 is an individual Newtonian potential for a point mass. Prior to that, Adrien-Marie Legendre analysed the expansion of the Newtonian potential in powers of $r = |\mathbf{x}|$ and $r_1 = |\mathbf{x}_1|$. He then discovered that if $r \leq r_1$ then

$$\frac{1}{|\mathbf{x}_j - \mathbf{x}|} = P_0 \cos(\gamma)\frac{1}{r_1} + P_1 \cos(\gamma)\frac{r}{r_1^2} + P_2 \cos(\gamma)\frac{r^2}{r_1^3} + \ldots \qquad \text{(Equation 5)}$$

where $\gamma$ is the angle between the vectors $\mathbf{x}$ and $\mathbf{x}_1$ and the functions $P_j$: $[-1, 1] \rightarrow \mathrm{R}$ are denoted as Legendre polynomials. Afterwards, Laplace investigated (in his 1782 memoir) these coefficients using spherical coordinates to represent the angle $\gamma$ between $\mathbf{x}_1$ and $\mathbf{x}$.

The name of "spherical harmonics" was first introduced in 1867 by William Thomson (Lord Kelvin) and Peter Guthrie Tait when they used solid spherical harmonics for these functions in their *Treatise on Natural Philosophy*. The solid harmonics were homogeneous polynomial solutions $\mathrm{R}^3 \rightarrow \mathrm{R}$ of Laplace's equation

$$\frac{\partial^2 u}{\partial x^2} + \frac{\partial^2 u}{\partial y^2} + \frac{\partial^2 u}{\partial z^2} = 0. \qquad \text{(Equation 6)}$$

Thomson and Tait derived Laplace's spherical harmonics by examining Laplace's equation in spherical coordinates. The term "Laplace's coefficients" was used by William Whewell to describe the particular system of solutions introduced along these lines, whereas others reserved this designation for the zonal spherical harmonics that had properly been introduced by Laplace and Legendre.

### *Analogy with Fourier series*

Any periodic function $f(\vartheta)$ with a period of $2\pi$ can be view, in an equivalent way, as a function defined on a circle with radius 1 and circumference $2\pi$. These functions, assume the same values every $2\pi$, therefore, they can be projected onto the unit circle (Figure S4A).

Additionally, any periodic function can be represented as Fourier series, a weighted sum of sines and cosines of different frequencies. Indeed, for any $2\pi$ periodic function $f(\vartheta)$, it is possible to find a set of constants (weights) $a_n$ and $b_n$ such that:

$$f(\vartheta) = \frac{a_0}{2} + \sum_{n=1}^{\infty} a_n \cos(n\vartheta) + b_n \sin(n\vartheta). \quad \text{(Equation 7)}$$

The study of Fourier series. i.e. the study of the way general functions may be represented or approximated by sums of simpler trigonometric functions (“basis functions”), is named Fourier analysis (Figure S4B).

Any function can then be approximated in terms of sines and cosines and its precision is given by the number of weights ($a_n$, $b_n$), and therefore basis function, used. This representation is exact only if an infinite numbers of basis functions is used (Kong et al., 2020). Since the basis function are sum of sines and cosines, and therefore periodic, they can also be represented as functions defined on a circle (*as explained before*).

In the case of spherical harmonics the concept is analogous. However, instead of having basis functions defined on a circle, spherical harmonic functions are defined on a sphere (i.e. they from a basis for $L_2(S^2)$). In the same way a periodic function can be projected on a circle (Figure S4A) it can also be projected on a sphere. With the Fourier analysis it is possible to convert any function defined on a circle into a weighted sum of sines and cosines of different frequencies. Similarly, any function defined on a sphere can be expressed as the weighted sum of the spherical harmonic functions $Y_l^m$. Specifically, given a function $f(\vartheta, \varphi)$, it is possible to find a series of coefficients $\mathbf{c}_l^m = (c_{lx}^m, c_{ly}^m, c_{lz}^m)$, such as:

$$f(\vartheta, \varphi) = \sum_{l=0}^{\infty} \sum_{m=-l}^{l} c_l^m Y_l^m(\vartheta, \varphi) \quad \text{(Equation 8)}$$

where:

$$Y_l^m(\vartheta, \varphi) = \sqrt{\frac{(2l+1)}{4\pi} \frac{(l-m)!}{(l+m)!}} P_l^m(\cos\vartheta)\ e^{im\varphi}, \quad \text{(Equation 9)}$$

and $P_l^m$ are the associated Legendre polynomials. In particular, $\mathbf{c}_l^m$ assume the form:

$$\begin{array}{ccc} \mathbf{c}_l^m & = & c_0^0 \\ & & c_1^{-1}\ c_1^0\ c_1^1 \\ & & c_2^{-2}\ c_2^{-1}\ c_2^0\ c_2^1\ c_2^2 \\ & & \ldots \end{array} \quad \text{(Equation 10)}$$

An example of the spherical harmonic $Y_1^0$ (degree $l = 1$ and order $m = 0$) is represented in Figure S4C. Often, however, spherical harmonics are not represented directly on the sphere but in the more common form depicted in Figure S4C (*right*). The two representations are fully equivalent, in Figure S4C (*left*) $Y_l^m$ is shown as a surface through all points on the sphere such as $\{\mathbf{x} | \|\mathbf{x}\|_2 = 1\}$ while in Figure S4C (*right*) it is represented as a surface through all points on the sphere such as $\{\mathbf{x} | f(\mathbf{x}) | \|\mathbf{x}\|_2 = 1\}$.

While the Fourier series has only one sum (Equation 7), in the case of spherical harmonics there are two (Equation 8). The index $l$ of the first one represents the “degree” of the function, and it can be seen as the equivalent of the frequency of the Fourier series. The index $m$ of the second sum constitutes the “order” and it represents the fact that for every degree $l$ there are $2l+1$ functions (differently from the Fourier series where there are 2 functions for every frequency $n$). Similarly to the constant term $\frac{a_0}{2}$ of the Fourier series, the spherical harmonics have, for $l = 0$ and $m = 0$, the constant term $c_0^0 Y_0^0$ which is a function that assumes the same value throughout the sphere.

Differently from the Fourier series, where each of the $n^{th}$ terms assume always the same form of sum of $\sin(n\vartheta)$ and $\cos(n\vartheta)$, the spherical harmonics are a more complex set of functions. The efficient calculation of the different values are indeed non-trivial to derive since they involve several functions which are themselves made of the derivatives of Legendre polynomials of varying degrees (*see* Arfken et al. [2013] for the complete derivation). As an informative example, we list here the first spherical harmonic functions, specifically for $l = 0, 1, 2$ and non-negative values of $m$ (*see* Arfken et al. [2013] for a more exhaustive table):

$$l = 0, \quad Y_0^0(\vartheta,\varphi) = \frac{1}{2}\frac{1}{\sqrt{\pi}}$$

$$l = 1, \quad \begin{cases} Y_1^1(\vartheta,\varphi) = -\frac{1}{2}\sqrt{\frac{3}{2\pi}}\sin\vartheta\ e^{i\varphi} \\ Y_1^0(\vartheta,\varphi) = \frac{1}{2}\sqrt{\frac{3}{\pi}}\cos\vartheta \end{cases}$$

$$l = 2, \quad \begin{cases} Y_2^2(\vartheta,\varphi) = \frac{1}{4}\sqrt{\frac{15}{2\pi}}\sin^2\vartheta\ e^{2i\varphi} \\ Y_2^1(\vartheta,\varphi) = -\frac{1}{2}\sqrt{\frac{15}{2\pi}}\sin\vartheta\cos\vartheta\ e^{i\varphi} \\ Y_2^0(\vartheta,\varphi) = \frac{1}{4}\sqrt{\frac{5}{\pi}}(3\cos^2\vartheta - 1) \end{cases} \quad \text{(Equation 11)}$$

In Figure S5 is shown the representation of the spherical harmonic functions for $l = 0, 1, 2, 3$ (Hill, 2020). For more information *see* Shafkat, 2021.

## Implementation

The method is written in Python 3 and depends on standard python packages (Harris et al., 2020).

All the mouse embryos were staged using the embryonic Mouse Ontogenetic Staging System (eMOSS) (Musy et al., 2018).

Surfaces of the data were extracted from the OPT scans using the marching cubes algorithm (Lorensen and Cline, 1987).

The alignment in space of the mouse limbs was done using the reference provided by eMOSS (Musy et al., 2018) and refined with the iterative closest point (ICP) algorithm (Besl and McKay, 1992).

Spherical harmonics transforms were made through the SHTools library (Wieczorek and Meschede, 2018). Specifically, we used the Python package *pyshtools* which intrinsically access to the Fortran-95 SHTOOLS library by use of Python wrapper functions. This package uses the fast Fourier transform (FFT) over latitude bands and exploits symmetry relations about the equator when calculating the associated Legendre functions which significantly decrease the computational time. It is therefore able to provide fast and accurate transforms up to spherical harmonic degree 2800. Here below, we briefly summarise how the spherical harmonic transformation and reconstruction are implemented in the package.

In order to obtain the spherical harmonic coefficients of a function, it is possible to show that, multiplying Equation 1 by $Y_{l'}^{m'}$ and integrating over space, they can be calculated by:

$$c_l^m = \frac{1}{4\pi}\int_{\Omega} f(\vartheta,\varphi)Y_l^m(\vartheta,\varphi)\ d\Omega \quad \text{(Equation 12)}$$

For calculating the spherical harmonic transform of a function, it is necessary to notice that Equation 12 can be written in a two-component vector notation where the two elements are for the cosine and sine spherical harmonic coefficient:

$$(C_l^m, S_l^m) = \frac{1}{4\pi}\int_0^{2\pi}\int_0^{\pi} f(\vartheta,\varphi)\overline{P}_l^m(\cos(m\varphi),\sin(m\varphi))\sin\varphi\ d\vartheta d\varphi. \quad \text{(Equation 13)}$$

Defining

$$\left(c_l^{m(j)}, s_l^{m(j)}\right) = \int_0^{2\pi} f(\vartheta_i,\varphi)(\cos(m\varphi),\sin(m\varphi))\ d\varphi \quad \text{(Equation 14)}$$

as intermediate variables, for a given co-latitude $\vartheta_j$ and degree $l$, all the angular order can be simultaneously calculated using a FFT of the function $f$. The spherical harmonic coefficients can therefore be calculated by replacing the integral over latitude in Equation 13 with a numerical quadrature rule as

$$(C_l^m, S_l^m) = \frac{1}{4\pi}\sum_{j=1}^{N} w_j\overline{P}_l^m\cos\vartheta_j\left(c_l^{m(j)}, s_l^{m(j)}\right) \quad \text{(Equation 15)}$$

where $w$ is the latitudinal weight and $N$ is the number of latitudinal points over which the integration is performed. It is possible to choose find weights $w_j$ and locations of the latitudinal sampling points $\vartheta_j$ to have an exact quadrature in Equation 15. In the SHTools package are implemented two quadrature rules: Gauss-Legendre and one based on the sampling theorem of Driscoll and Healy (*see* Wieczorek and Meschede [2018] *for more information*). In both cases the quadrature is exact only if the spherical harmonic degree is fixed to a maximum, i.e. $l = l_{\max}$.

To reconstruct a function from its spherical harmonic coefficients, the implementation starts by using the separate variables $C_l^m$ and $S_l^m$ for rewriting Equation 1 as

$$f(\vartheta,\varphi) = \sum_{m=0}^{L}(a_m(\vartheta)\cos(m\varphi) + b_m(\vartheta)\sin(m\varphi)) \quad \text{(Equation 16)}$$

where

$$(a_m(\vartheta), b_m(\vartheta)) = \sum_{l=m}^{L}(C_l^m, S_l^m)\overline{P}_l^m \cos\vartheta \quad \text{(Equation 17)}$$

and $L = l_{max}$. For increasing the speed of calculation, SHTools evaluates the function $f$ on a series of grid nodes all at the same time using an inverse FFT (*see* Wieczorek and Meschede [2018] *for the details of the FFT calculation*). The slowest part of the process is the calculation of the coefficients $a_m$ and $b_m$ which depend on the associated Legendre functions. They are calculated using using standard three-term recursion relations over adjacent spherical harmonic degrees, specifically, for a given co-latitude, the sectoral term $\overline{P}_{mm}$ is first calculated using an analytic equation, and then $\overline{P}_{lm}$ is calculated for all values of $l > m$ (*see* Wieczorek and Meschede [2018] *for more details*).

### Whole-mount antibody staining

Embryos were dehydrated with methanol and left overnight at -20° C. Whole-mount immunochemistry was performed according to standard protocols. Embryos were rehydrated, permeabilized in PBS plus 0.5% Triton X-100 (Sigma), left 2h in blocking solution (90% PBST (0.1% Triton X-100 in PBS)/10% normal goat serum) and incubated overnight at 4° C with anti-Myosin heavy chain, sarcomere (MHC) antibody (Developmental Studies Hybridoma Bank, MF20; 1:10). Embryos were washed in PBST four times for 4 hours and incubated overnight with biotin goat anti-mouse IgG (Jackson ImmunoResearch ref. 115-066-071; 1:500) and then with streptavidin-Cy3 (Jackson ImmunoResearch ref. 016-160-084; 1:500). After washing in PBST four times for four hours, embryos were stored in PBST 0.01% sodium azide and subjected to OPT scanning to obtain images.

### Optical projection tomography

Optical projection tomography (OPT) imaging (Sharpe et al., 2002) was used to acquire 2D images and obtain 3D reconstructions. MF20 immunostained samples were embedded in 1% low melting point agarose (Sigma), dehydrated in 100% methanol and cleared in BABB (1 volume benzyl alcohol : 2 volumes benzyl benzoate). Samples were scanned at intermediate resolution (512x512 pixels) in the Bioptonics OPT scanner using Skyscan software (Bioptonics, MRC Technology). The GFP filter (425/40nm, 475nm LP) was used to scan whole embryo anatomy, while the Cy3 filter (545/30nm, 610/75nm) was used to image the heart. OPT scans were reconstructed using NRecon software (SkyScan) and analyzed using the Bioptonics Viewer software.

## QUANTIFICATION AND STATISTICAL ANALYSIS

The comparison between the reconstructed limbs and the original data shown in Figures 2C and 4A were computed applying the signed distance using the angle weighted pseudonormal (Baerentzen and Aanaes, 2005) implemented in *Vedo* Musy et al., 2022).

The bending of the flanks to make them match with the reference limbs shown in Figure 3C was done by using a linear fit of $\alpha$ as a reference, and to apply a bending transform onto the shape data using *thin plate splines* (Bookstein, 1989) (implemented in *Vedo* (Musy et al., 2022)), which is a non-linear transformation based on a physical analogy involving the bending of a rigid material. The limbs were not affected by this transformation, only the non aligned flanks were bent.

The alignment in space of the mouse hearts was done manually applying a 4x4 linear rigid transformation using the interactive capabilities of *Vedo* (Musy et al., 2022).

All the visualisations and animations were produced using *Vedo* (Musy et al., 2022).

## ADDITIONAL RESOURCES

In order to better-appreciate the results of this study, additional movies, 3D interactive views and further information can be found in the project's web-page → https://vedo.embl.es/fearless.

**Developmental Cell, Volume *57***

## Supplemental information

# 4D reconstruction of murine developmental trajectories using spherical harmonics

**Giovanni Dalmasso, Marco Musy, Martina Niksic, Alexandre Robert-Moreno, Claudio Badía-Careaga, Juan Jose Sanz-Ezquerro, and James Sharpe**

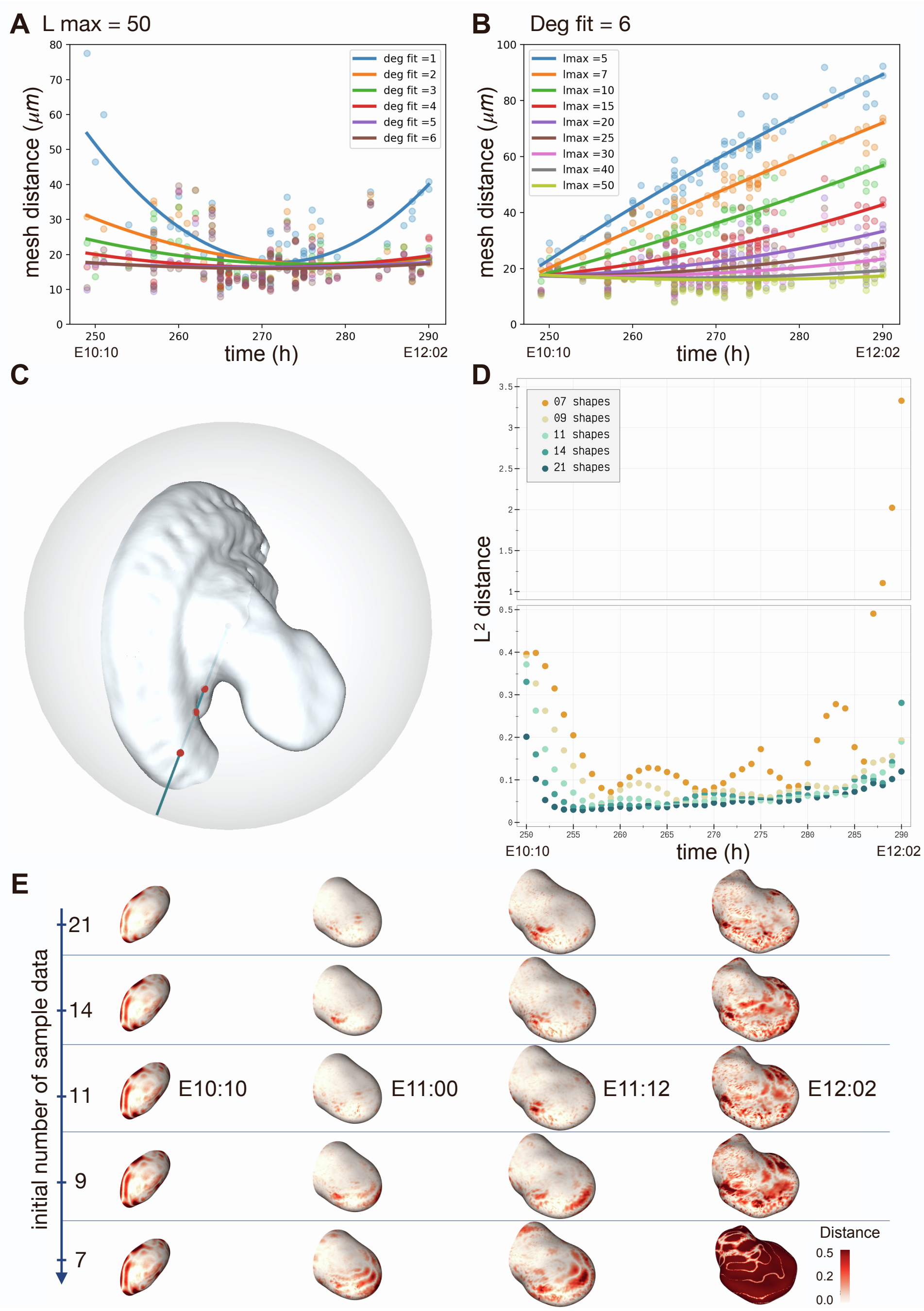


**Figure S1: Assessing the degree of spherical harmonics expansion**, related to Figure 1.

**A** - Comparison of different reconstructions according to mesh distances fixing the value of $l_{\max} = 50$ and varying the degree of the spline (from 1 to 6) interpolating the spherical harmonics coefficients.

**B** - Comparison of different reconstructions according to mesh distances fixing the degree's value of the spline interpolating the spherical harmonics coefficients and the value of $l_{\max}$ (5, 7, 10, 15, 20, 25, 30, 35, 40, 45, 50).

**C** - Representative example of a surface intersected three times (red dots) by a radius of a sphere which circumscribes it.

**D** - $L^2$ distance between sets of reconstructed and original surfaces reducing the total number of the initial set of data (i.e: 21, 14, 11, 9, 7).

**E** - Sets of reconstructed surfaces reducing the total number of the initial set of data (i.e: 21, 14, 11, 9, 7) at four different developmental stages (i.e: E10:10, E11:01, E11:12, E12:10). The color map shows the distance between the reconstructed meshes and the original data.

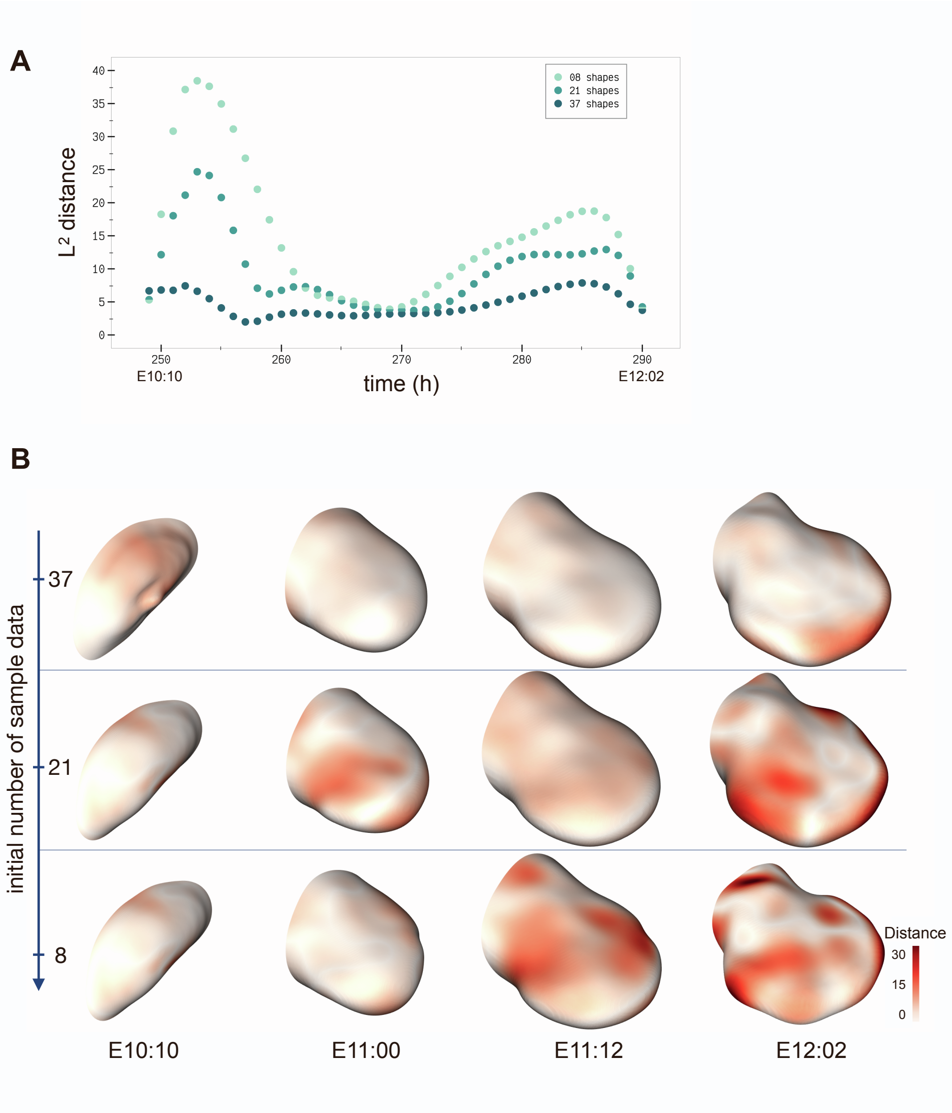


**Figure S2: Assessing the degree of spherical harmonics expansion**, related to Figure 1.
**A** - $L^2$ distance between sets of reconstructed and original surfaces reducing the total number of the initial set of data (i.e: 37, 21, 8).
**B** - Sets of reconstructed surfaces reducing the total number of the initial set of data (i.e: 37, 21, 8) at four different developmental stages (i.e: E10:10, E11:01, E11:12, E12:10). The color map shows the distance between the reconstructed meshes and the original data.

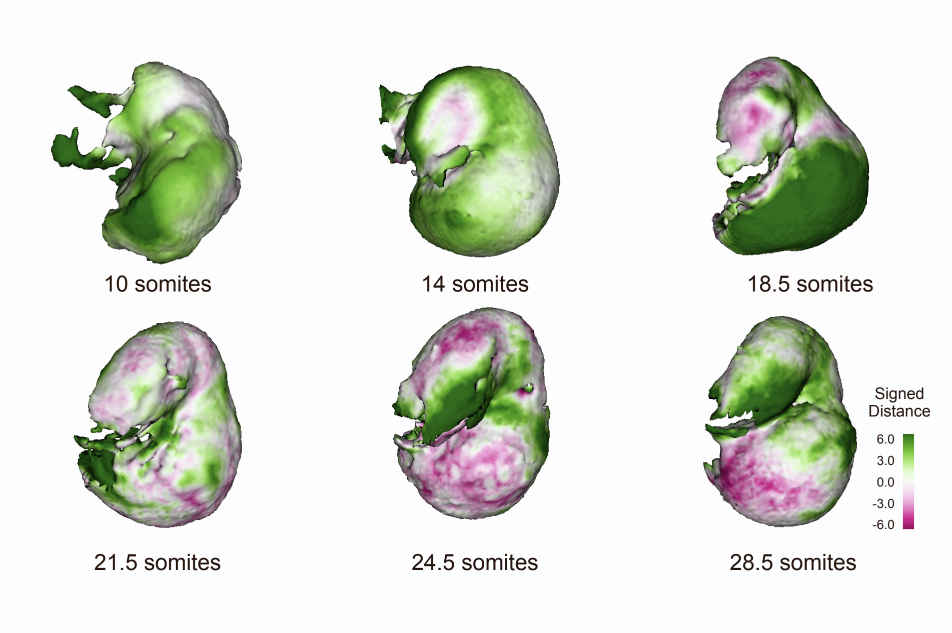


**Figure S3: Precision of the reconstruction**, related to Figure 5.
The heart reconstruction at representative developmental time points color map representing the signed distance between the reconstruction and the original surface.

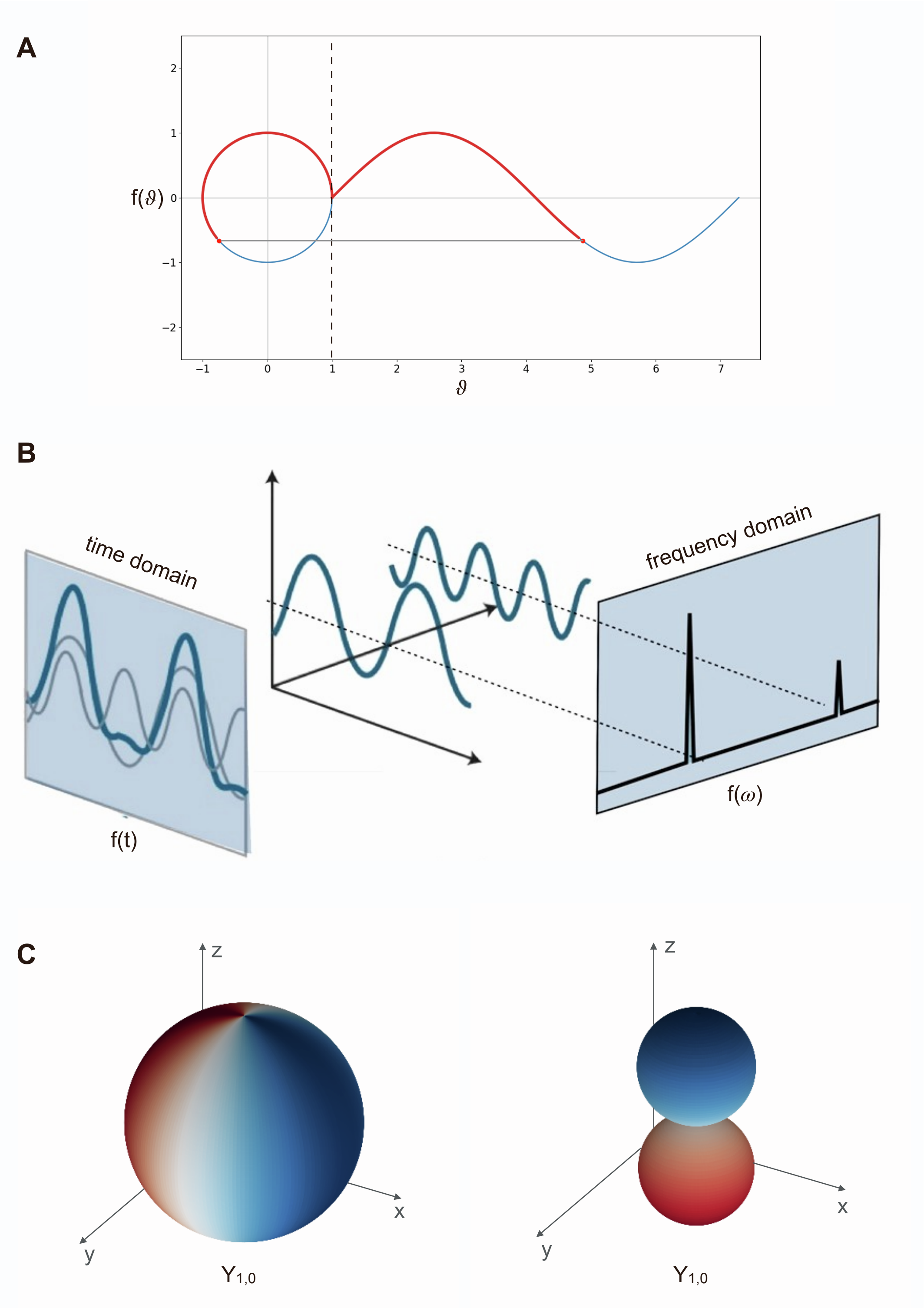


**Figure S4: Spherical harmonics and Fourier expansion**, related to STAR Methods.
**A** Representation of the function $\sin\vartheta$ and its projection onto the unit circle.
**B** Schematic representation of a function $f(t)$ in the time domain, its expression given by the sum of basis functions and its Fourier transform $f(\omega)$ in the frequency domain (*modified from* Gendler (2017)).
**C** Two equivalent representations of the spherical harmonic $Y_1^0$ of degree $l = 1$ and order $m = 0$.

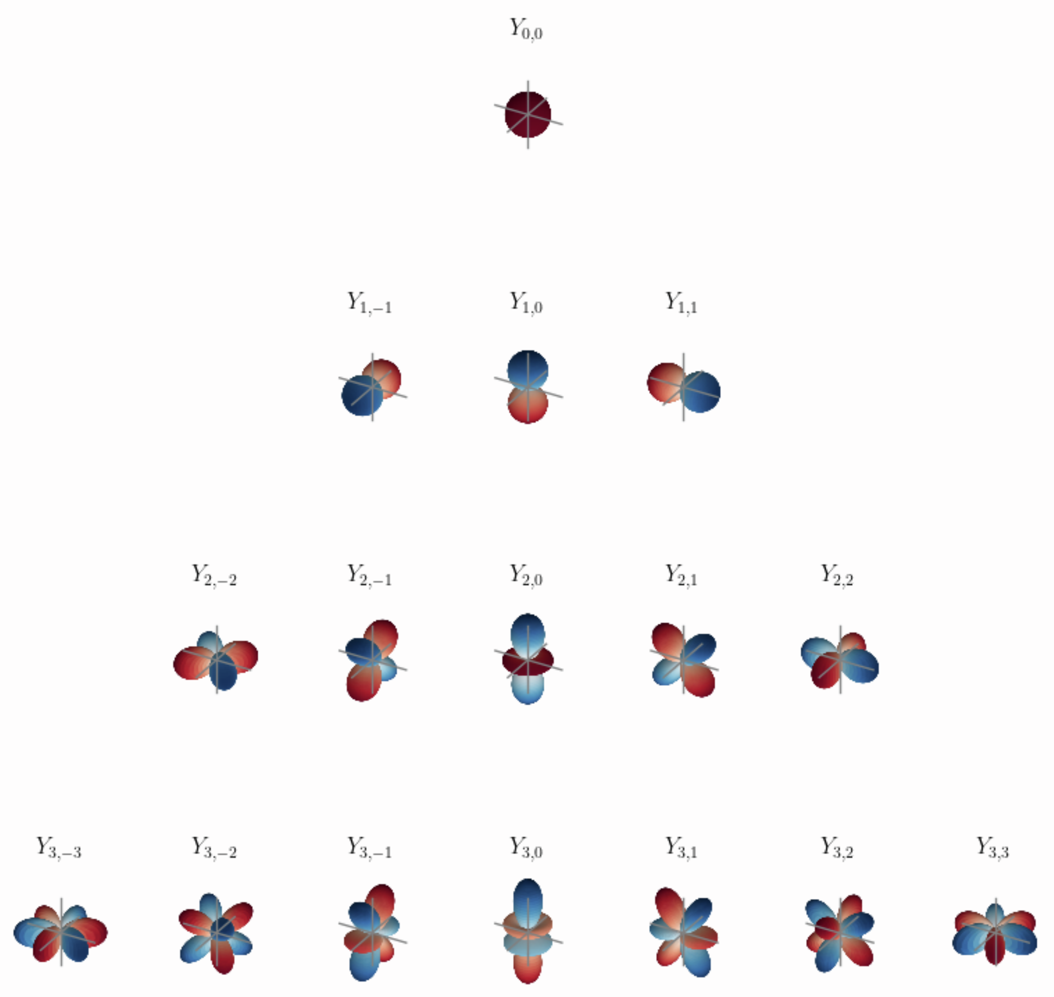


**Figure S5: Hierarchical tree of spherical harmonics expansion**, related to STAR Methods. Representations of spherical harmonic functions for $l = 0, 1, 2, 3$.
